# Temperature sensitivity of detrital photosynthesis

**DOI:** 10.1093/aob/mcad167

**Published:** 2023-12-24

**Authors:** Luka Seamus Wright, Taylor Simpkins, Karen Filbee-Dexter, Thomas Wernberg

**Affiliations:** Oceans Institute, University of Western Australia, Perth,Australia; School of Biological Sciences, University of Western Australia, Perth,Australia; Oceans Institute, University of Western Australia, Perth,Australia; School of Biological Sciences, University of Western Australia, Perth,Australia; Oceans Institute, University of Western Australia, Perth,Australia; School of Biological Sciences, University of Western Australia, Perth,Australia; Institute of Marine Research, His, Norway; Oceans Institute, University of Western Australia, Perth,Australia; School of Biological Sciences, University of Western Australia, Perth,Australia; Institute of Marine Research, His, Norway

**Keywords:** Brown algae, climate change mitigation, CO_2_, Laminariales, detrital dynamics, macroalgae, macroalgal carbon dioxide removal, Phaeophyceae, ocean warming, photoacclimation, photophysiology, temperature tolerance

## Abstract

**Background and Aims:**

Kelp forests are increasingly considered blue carbon habitats for ocean-based biological carbon dioxide removal, but knowledge gaps remain in our understanding of their carbon cycle. Of particular interest is the remineralization of detritus, which can remain photosynthetically active. Here, we study a widespread, thermotolerant kelp (*Ecklonia radiata*) to explore detrital photosynthesis as a mechanism underlying temperature and light as two key drivers of remineralization.

**Methods:**

We used meta-analysis to constrain the thermal optimum (*T*_opt_) of *E. radiata*. Temperature and light were subsequently controlled over a 119-day *ex situ* decomposition experiment. Flow-through experimental tanks were kept in darkness at 15 °C or under a subcompensating maximal irradiance of 8 µmol photons m^−2^ s^−1^ at 15, 20 or 25 °C. Photosynthesis of laterals (analogues to leaves) was estimated using closed-chamber oxygen evolution in darkness and under a saturating irradiance of 420 µmol photons m^−2^ s^−1^.

**Key Results:**

*T*_opt_ of *E. radiata* is 18 °C across performance variables (photosynthesis, growth, abundance, size, mass and fertility), life stages (gametophyte and sporophyte) and populations. Our models predict that a temperature of >15 °C reduces the potential for *E. radiata* detritus to be photosynthetically viable, hence detrital *T*_opt_ ≤ 15 °C. Detritus is viable under subcompensating irradiance, where it performs better than in darkness. Comparison of net and gross photosynthesis indicates that elevated temperature primarily decreases detrital photosynthesis, whereas darkness primarily increases detrital respiration compared with optimal experimental conditions, in which detrital photosynthesis can persist for ≥119 days.

**Conclusions:**

*T*_opt_ of kelp detritus is ≥3 °C colder than that of the intact plant. Given that *E. radiata* is one of the most temperature-tolerant kelps, this suggests that photosynthesis is generally more thermosensitive in the detrital phase, which partly explains the enhancing effect of temperature on remineralization. In contrast to darkness, even subcompensating irradiance maintains detrital viability, elucidating the accelerating effect of depth and its concomitant light reduction on remineralization to some extent. Detrital photosynthesis is a meaningful mechanism underlying at least two drivers of remineralization, even below the photoenvironment inhabited by the attached alga.

## INTRODUCTION

Fundamental knowledge of kelp carbon cycling is required before kelp forests can be included into blue carbon frameworks and accredited for their climate-mitigating role in ocean-based biological carbon dioxide removal (CDR) (Krause-Jensen and Duarte, 2016; Pessarrodona *et al.*, 2023). Kelps are among the most productive plants on Earth (Pessarrodona *et al.*, 2022*b*), but current empirical estimates of carbon sequestration are not sufficiently precise to predict CDR by a given area of kelp forest (Queirós *et al.*, 2019; Wright *et al.*, 2022). In part, this is attributable to the predominantly allochthonous fate of kelp carbon. Key phases of the carbon cycle that need to be understood thoroughly are carbon assimilation (photosynthesis), export (erosion, abscission, dislodgement and transport), remineralization (herbivory, decomposition and physical degradation) and sequestration (burial and retention in water). Substantially more data exist on global kelp carbon assimilation (Duarte *et al.*, 2022; Pessarrodona *et al.*, 2022*b*) and export (Pessarrodona *et al.*, 2023) than on remineralization and sequestration (Queirós *et al.*, 2019; Ravaglioli *et al.*, 2019; Wright *et al.*, 2022). Specifically, drivers of remineralization (the “what”) and their underlying mechanisms (the “how” and “why”) are scarcely understood. This level of understanding is important for assessments of kelp CDR because remineralization plays a key role in determining carbon fate, manifesting during all phases of the carbon cycle (Arnosti, 2011; Pessarrodona *et al.*, 2023).

Drivers of remineralization are better understood than mechanisms. Drivers are either intrinsic or extrinsic. By nature, intrinsic drivers are taxon specific and are therefore useful tools when estimating inter-taxa differences in CDR, whereas extrinsic drivers are environmental, affecting all taxa, and are thus better suited for spatial and temporal CDR estimation. Consequently, both are essential for reliable context-specific prediction of CDR. The best-studied intrinsic driver is elemental stoichiometry. Higher carbon-nutrient ratios (Enríquez *et al.*, 1993) and carbon concentrations (Filbee-Dexter *et al.*, 2022) and lower nutrient concentrations (Cebrián and Lartigue, 2004) are predicted to slow remineralization (but see Wright *et al.*, 2022). There is also evidence for an inhibitory effect of chemical defences, such as phenols, iodine and reactive oxygen species (Küpper *et al.*, 2002; de Bettignies *et al.*, 2020; Wright *et al.*, 2022), and structural recalcitrance (Trevathan-Tackett *et al.*, 2015; Pedersen *et al.*, 2021; Wright *et al.*, 2022). Among the extrinsic drivers, elevated temperature (Litchfield *et al.*, 2020; Frontier *et al.*, 2021*a*; Pedersen *et al.*, 2021; Filbee-Dexter *et al.*, 2022) and reduced light (Frontier *et al.*, 2021*a*, *b*; Pedersen *et al.*, 2021) are predicted to strongly accelerate remineralization (but see Rothäusler *et al.*, 2011; Xiao *et al.*, 2015 on photodegradation). However, other extrinsic drivers, such as pollution (Litchfield *et al.*, 2020) and oxygen (Pedersen *et al.*, 2021), can be more important in some cases. Knowing general drivers alone is not sufficient to understand the fate of kelp carbon because there are always nuances and exceptions (Pedersen *et al.*, 2021; Wright *et al.*, 2022). Only when the underlying mechanisms are understood is robust prediction of CDR possible.

The mechanisms of remineralization are thought to be predominantly extrinsic in nature because remineralization is either a trophic or a physical process with some external driving force. For instance, heterotrophic activity is assumed to be the main mechanism behind diverse drivers of remineralization, such as elemental stoichiometry, temperature, oxygen and pollution (Litchfield *et al.*, 2020; Frontier *et al.*, 2021*a*; Pedersen *et al.*, 2021; Filbee-Dexter *et al.*, 2022). This assumption is founded on our general understanding of the lability of nutrient-rich compounds (Enríquez *et al.*, 1993; Cebrián and Lartigue, 2004; Arnosti, 2011; Sosik and Simenstad, 2013) and the temperature dependence of heterotrophic metabolism (Yvon-Durocher *et al.*, 2012; Wilken *et al.*, 2013; Smale *et al.*, 2017). Conversely, the mechanism underlying the effect of light on remineralization, with the exception of photodegradation, is assumed to be intrinsic, i.e. physiological. Studies comparing decomposition rates of prekilled (Birch *et al.*, 1983; Brouwer, 1996) or aged (de Bettignies *et al.*, 2020) with that of fresh macroalgal detritus always found the latter to decompose more slowly. Such indirect proof of detrital metabolic activity eventually led to the first direct evidence and definition of detrital photosynthesis (Frontier *et al.*, 2021*b*; Wright *et al.*, 2022). Detrital photosynthesis has been demonstrated directly or indirectly in the kelps *Macrocystis pyrifera* (Macaya *et al.*, 2005; Rothäusler *et al.*, 2011, 2018), *Durvillaea antarctica* (Tala *et al.*, 2013, 2019; Fraser *et al.*, 2018), *Saccharina latissima* (Filbee-Dexter *et al.*, 2022; Wright and Kregting, 2023) and the three Northeast Atlantic *Laminaria* species (de Bettignies *et al.*, 2020; Frontier *et al.*, 2021*a*, *b*; Pedersen *et al.*, 2021; Wright and Foggo, 2021; Filbee-Dexter *et al.*, 2022; Wright *et al.*, 2022; Wright and Kregting, 2023) by means of measuring oxygen production, pigmentation, chlorophyll fluorescence, growth and/or fertility. Understanding the photophysiology of detritus is important because it can decelerate (Birch *et al.*, 1983; Brouwer, 1996; de Bettignies *et al.*, 2020) or even counterbalance and reverse (Frontier *et al.*, 2021*a*, *b*; Pedersen *et al.*, 2021; Filbee-Dexter *et al.*, 2022; Wright *et al.*, 2022) remineralization. Although detrital photosynthesis has been suggested as the mechanism behind the light driver (Frontier *et al.*, 2021*a*, *b*), this process deserves validation. Furthermore, although temperature and light have been investigated in this context, current evidence only links light to detrital photosynthesis (Frontier *et al.*, 2021*a*). The temperature response of detrital photosynthesis and, consequently, its potential contribution to the temperature driver therefore remain unknown.

Here we aim to gain a better understanding of the mechanisms at the root of temperature and light as drivers of remineralization. Like photosynthesis in the intact plant, detrital photosynthesis must have temperature and light optima. Estimates of such photophysiological parameters will enable more accurate prediction of kelp carbon remineralization, hence CDR, under various temperature and light regimes. This is relevant in the context of detrital transport through waterbodies varying in temperature and light, either spatially, owing to depth and location, or temporally, owing to ocean warming (Filbee-Dexter *et al.*, 2022) and coastal darkening (Blain *et al.*, 2021). A first step towards understanding general optima is to focus on extremes. We therefore studied the thermal response of detrital photosynthesis in the exceptionally thermotolerant kelp *Ecklonia radiata*, a species for which detrital photosynthesis has never been measured, at subcompensating irradiance compared with darkness. Our objective was to constrain the thermo- and photoacclimation limits of *E. radiata* detritus, because ocean warming has been speculated to diminish CDR (Filbee-Dexter *et al.*, 2022; Wright *et al.*, 2022), and the detritus of interest to CDR is that transported to the deepest depths (Krause-Jensen and Duarte, 2016; Pessarrodona *et al.*, 2023), where light is limiting.

## MATERIALS AND METHODS

### Model species

**Fig. 1.**
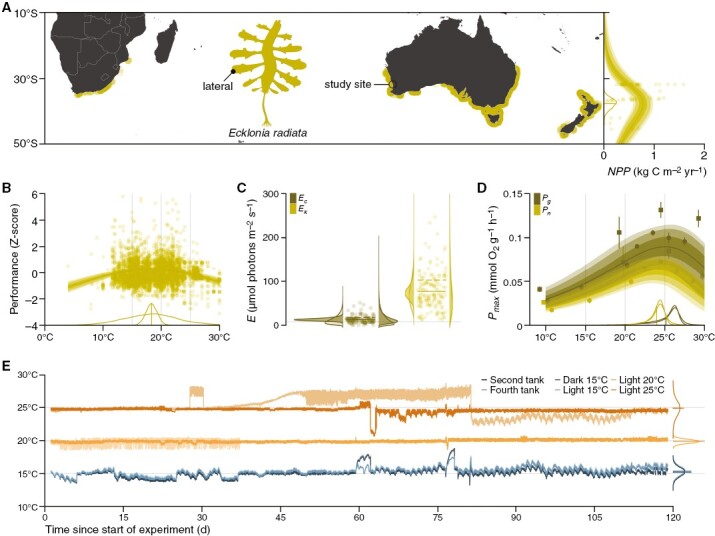
Experimental treatments in the context of *Ecklonia radiata* temperature and light tolerance. (**A**) Geography of the study site, species distribution (GBIF, 2023; OBIS, 2023) and net primary production (Pessarrodona *et al.*, 2022*a*). The map is oriented north, based on the WGS 84 coordinate reference system and rendered according to the equirectangular projection. Net primary production (*NPP*) is modelled with the peaked Arrhenius equation (Medlyn *et al.*, 2002; Terada *et al.*, 2016). Lines and intervals are means and 50, 80 and 90 % posterior probability intervals for the mean prediction. The posterior probability distribution of *T*_opt_ and its mean are plotted below the curve. Note that one data point (36°S, 5.5 kg C m^−2^ year^−1^) is excluded from the plot, but not from the model. (**B**) Temperature response of standardized performance (photosynthesis, growth, abundance, size, mass and fertility) across life stages (gametophyte and sporophyte) and populations (Wright, 2023). Performance is modelled with the Gaussian function. Lines and intervals are means and 50, 80 and 90 % posterior probability intervals for the mean prediction. Posterior probability distributions of *T*_opt_ and their mean are plotted for the given data (narrow) and predicting the outcome of a new study (wide) and rescaled relative to one another. Note that several data points above a *z*-score of 6 are excluded from the plot, but not from the model. Vertical grey lines mark experimental treatment targets. (**C**) Compensation (*E*_c_) and saturation (*E*_k_) points for sporophyte photosynthesis (Fairhead and Cheshire, 2004; Miller *et al.*, 2006; Staehr and Wernberg, 2009; Rodgers *et al.*, 2015; Rodgers and Shears, 2016; Blain and Shears, 2019, 2020; Randall *et al.*, 2019; Blain *et al.*, 2020). Given that irradiance cannot attain negative values, *E*_c_ and *E*_k_ were modelled using the gamma distribution. Posterior probability distributions of *E*_c_ and *E*_k_ delimit their means (dark) or full range of observations (light) and predict given data (left) or the outcome of a new study (right). Lines across data points are means for the left (solid) and right (dashed) distributions. The horizontal grey line marks the experimental treatment target. (**D**) Temperature response of net (*P*_n_) and gross (*P*_g_) light-saturated sporophyte photosynthesis at our study site (**A**, 31.789367°S, 115.679017°E) stratified by study (■: Staehr and Wernberg, 2009; ● : Wernberg *et al.*, 2016). Photosynthesis is modelled using the peaked Arrhenius equation (Medlyn *et al.*, 2002; Terada *et al.*, 2016). Lines and intervals are means and 50, 80 and 90 % posterior probability intervals for the mean prediction. Posterior probability distributions of *T*_opt_ and their mean are plotted for the given data (narrow) and predicting the outcome of a new study (wide). Point ranges denote mean ± frequentist standard error of the mean (comparable to Bayesian standard deviation of the posterior probability distribution of the mean). Note that point ranges are *x*-shifted relative to one another to avoid overplotting. Vertical grey lines mark experimental treatment targets. (**E**) Experimental temperature in the second and fourth tank of each treatment. Probability distributions across the second and fourth tanks delimit the means (narrow) or full range of observations (wide) and are rescaled relative to one another. Horizontal grey lines mark the temperature treatment target (cf. **B**–**D**). For details on meta-analyses, see github.com/lukaseamus/detrital-tolerance.

*Ecklonia radiata* is widely distributed in the warm temperate Southern Hemisphere, with optimal sporophyte net primary production across continents (0.8 ± 0.12 kg C m^−2^ year^−1^, mean ± standard deviation of the mean) at 38 ± 1.6°S (Fig. 1A) and optimal performance across measures (photosynthesis, growth, abundance, size, mass and fertility), life stages (gametophyte and sporophyte) and populations at 18 ± 0.98 °C (±4.9 °C when predicting the outcome of a new study) (Fig. 1B). Like other low-latitude kelps (Dominik and Zimmerman, 2006; Graham *et al.*, 2007; Marins *et al.*, 2014; García-Sánchez *et al.*, 2016; Buglass *et al.*, 2022), *E. radiata* also forms mesophotic forests (Marzinelli *et al.*, 2015) and can acclimate to low light (Miller *et al.*, 2006). Its compensation point (*E*_c_) of 13 µmol photons m^−2^ s^−1^ (18 µmol photons m^−2^ s^−1^ when predicting the outcome of a new study) (Fig. 1C) is comparable to that of congeners (Terada *et al.*, 2016) and other infralittoral kelps (Davison *et al.*, 1991; Andersen *et al.*, 2013) and the saturation point (*E*_k_) of circalittoral/mesophotic kelps (Dominik and Zimmerman, 2006; García-Sánchez *et al.*, 2016). At our study site (Fig. 1A), the thermal optima (*T*_opt_) for sporophyte net (65 ± 9.2 µmol O_2_ g^−1^ dry mass h^−1^) and gross (93 ± 15 µmol O_2_ g^−1^ dry mass h^−1^) photosynthesis lie at 24 ± 1.1 °C (±1.7 °C when predicting the outcome of a new study) and 25 ± 1.7 °C (±2.5 °C when predicting the outcome of a new study), respectively (Fig. 1D; Staehr and Wernberg, 2009; Wernberg *et al.*, 2016). The considerable difference from the overall optimum of 18 °C (Fig. 1B) might be partly attributable to higher temperature tolerance of sporophytes in comparison to gametophytes (Wright *et al.*, 2022; but see Veenhof *et al.*, 2023) but is likely mostly attributable to the short temperature treatment exposure period during photosynthesis measurements (Staehr and Wernberg, 2009; Wernberg *et al.*, 2016; cf. Xiao *et al.*, 2015). We therefore expected *T*_opt_ for *E. radiata* detritus to lie at or above 18 °C. At our study site, *E*_c_ is known to increase from 16 µmol photons m^−2^ s^−1^ at 10 °C to 44 µmol photons m^−2^ s^−1^ at 30 °C (Staehr and Wernberg, 2009), hence the light minimum for detrital photosynthesis is predicted to lie at or above 13 µmol photons m^−2^ s^−1^. Accordingly, we chose 15, 20 and 25 °C as temperatures that span the expected *T*_opt_ and 8 µmol photons m^−2^ s^−1^ as a maximal irradiance (*E*_max_) with a 77 % probability of lying below *E*_c_ (Fig. 1B–D).

### Decomposition experiment

We designed an *ex situ* litterbag experiment (cf. Frontier *et al.*, 2021*a*, *b*; Pedersen *et al.*, 2021) to tease apart the influences of temperature and light as drivers of decomposition. These variables were explored through four categorical treatments designed to be representative of 15 °C in the dark (hereinafter dark treatment) and of 15, 20 and 25 °C, each at *E*_max_ = 8 µmol photons m^−2^ s^−1^ (hereinafter 15, 20 and 25 °C treatments). Four 16-L aquaria were allocated to each treatment. Each aquarium was supplied with flow-through seawater (32 L h^−1^) connected to one temperature-controlled header tank per temperature treatment that maintained inflowing seawater at 2 °C below the target temperature (2000 L; HC-2200BH 2 HP, Hailea Group Co. Ltd, Raoping, China). The temperature of each aquarium was also maintained independently by installing a 1000-W submersible twin heater that was turned on or off through an autonomous system (Touch Controller Kit, Aquatronica s.r.l., Reggio Emilia, Italy) when the temperature dropped below or above 1 °C of target temperature. Aquaria were equipped with a circulation pump (3000 L h^−1^; JVP-120, Sunsun Group Co. Ltd, Zhoushan, China) and irradiated with LED lights on a parabolic 12-h-light-12-h-dark cycle, peaking at *E*_max_, or covered with black plastic containers, depending on the treatment. Daily irradiance approximately increased from 0 to 4 µmol photons m^−2^ s^−1^ at 5:00, then to 8 µmol photons m^−2^ s^−1^ at 12:00, then decreased to 4 µmol photons m^−2^ s^−1^ at 16:00, and from 4 to 0 µmol photons m^−2^ s^−1^ at 17:00. The exact temperature and peak photon flux density were recorded throughout the experiment with temperature loggers (HOBO^®^ Pendant, Onset, Bourne, MA, USA) and a full spectrum underwater quantum meter (MQ-510, Apogee Instruments, Logan, UT, USA), respectively. Realized experimental treatments were 15 ± 0.67, 15 ± 0.66, 20 ± 0.3 and 25 ± 1.2 °C (mean ± standard deviation) for dark and light 15, 20 and 25 °C, respectively (Fig. 1E), and 7.9 ± 0.72 (*n* = 16), 8 ± 0.92 (*n* = 20) and 7.8 ± 0.97 (*n* = 20) µmol photons m^−2^ s^−1^ for 15, 20 and 25 °C, respectively.

Eighty litres of sediment was collected from 1-m depth in Watermans Bay, Western Australia (31.852559°S, 115.750848°E) on 23 June 2022, sieved (3-mm diameter) and added to each tank to a height of ~5 cm (~5 L). On 26 June, 40 mature *E. radiata* sporophytes were collected from ~10 m depth in the Three Mile Reef kelp forest in Marmion Marine Park, Western Australia (31.789367°S, 115.679017°E). Within 5 h, sporophytes were transported on ice to the Indian Ocean Marine Research Centre in Watermans Bay and stored at 10 °C for 48 h. On 28 June, three laterals (leaflike lamina protuberances; Fig. 1A) were excised from each sporophyte at the narrow lateral-lamina interface (Fig. 1A) to minimize wound perimeter, which was consistently ~4 cm. Starting from the side of the cut, laterals were trimmed to a blotted mass of 10 ± 0.1 g (0.01-g accuracy; 440-33N, Kern & Sohn GmbH, Balingen, Germany) and placed individually in mesh bags (3-mm diameter) with an inert aquarium pebble to weigh them down onto the sediment surface and mimic benthic detritus. Hence, detritus is defined here as excised laterals in contact with sediment under the exclusion of macrofaunal detritivores. All mesh bags were stored overnight in a 220-L holding tank supplied with ambient (~20 °C) flow-through seawater. On 29 June, initial photosynthesis of five randomly sampled replicate laterals was quantified (see *Measurement of photosynthesis*), and the experiment was commenced by haphazardly removing mesh bags from the holding tank and placing seven in each of the 16 experimental tanks. By this time, the sediment microbiome had acclimated to the experimental conditions for 6 days. A single mesh bag containing a single lateral was sampled destructively from each tank for detrital photosynthesis measurement after 6, 13, 27, 37, 82 and 119 days, completing the experiment on 26 October, when almost all detrital tissue had decomposed.

### Measurement of photosynthesis

Maximal net photosynthesis (*P*_n_) and respiration (*R*) were measured in a 20 °C controlled temperature room using non-invasive, closed-chamber oxygen (O_2_) evolution (cf. Wright *et al.*, 2022) on the day of sampling. Gross photosynthesis (*P*_g_) was subsequently derived from *P*_n_ and *R* by addition (see *Data analysis and visualization*). Laterals, trimmed as required (2.4 ± 0.84 g blotted mass, 0.35 ± 0.16 g dry mass by conversion, mean ± standard deviation, *n* = 57, 0.005-g accuracy; Highland^®^, Adam Equipment Co. Ltd, Milton Keynes, UK), were incubated alongside a seawater blank in sealed 175-mL glass jars filled with oxygenated seawater (freshly collected from the flow-through system), equipped with magnetic stir bars and self-adhesive planar O_2_ sensor spots (SP-PSt3-SA-NAU-D5-YOP, PreSens Precision Sensing GmbH, Regensburg, Germany) and placed on a magnetic stirrer. Air was excluded from incubation chambers by closing their rubber seal underwater after thoroughly removing bubbles. A photon flux density with 98 % probability of being saturating (420 ± 19 µmol photons m^−2^ s^−1^, mean ± standard deviation, *n* = 40, cf. *E*_k_ in Fig. 1C; MQ-510, Apogee Instruments, Logan, UT, USA) was provided by an LED light (Zeus, Ledzeal, Shenzhen Topline Lighting Technology Co. Ltd, Shenzhen, China). To achieve the dark conditions required to detect respiration, the magnetic stirrer was covered with a black plastic container. The O_2_ meter was calibrated using anoxic (1 % w/v Na_2_SO_3_) and air-saturated (bubbled with air) ultrapure water.

Dissolved O_2_ (micromolar) was measured fibre-optically through the glass every 10 s over ~5 min (30 measurements) with a four-channel O_2_ meter (OXY-4 SMA G2, PreSens Precision Sensing GmbH, Regensburg, Germany) after initial monitoring to ensure that the short measurement period was representative. All measurements were corrected for incubation temperature (18 ± 0.38 °C, mean ± standard deviation), pressure (1022 ± 4.3 hPa) and salinity (35 ± 0.68 ‰) using a single temperature dipping probe connected to the first channel of the built-in temperature sensor of the O_2_ meter and placed in a fifth 175-mL jar filled with seawater at the same time as the four others, the built-in pressure sensor of the O_2_ meter and a hand-held refractometer, respectively. When measurement series were longer than 30, they were truncated by removing data from either the start or the end. The decision on whether to truncate the start or the end was based on minimization of measurement error and maximization of slope similarity in comparison to the untruncated series. If both optima conflicted, maximization of slope similarity was prioritized in the trade-off.

Initially, respiration was measured after photosynthesis to account for potential elevation of respiration during and after incubation under irradiance. However, rather than elevated O_2_ consumption after irradiance, O_2_ production was maintained in the dark following irradiance under saturating photon flux density, and O_2_ consumption only became evident after a period of >10 min in the dark. Conversely, O_2_ production commenced after dark incubation as soon as the kelp was irradiated. To streamline the experiment (up to six measurement rounds had to be conducted in a single day) and because there is evidence that measurement sequence does not matter (Miller III and Dunton, 2007), most measurements of respiration were therefore taken before the measurements of photosynthesis. This substantially reduced the required waiting time between measurements and seemingly did not affect respiration rates.

The described O_2_ evolution method only allows measurement if the sample is intact. O_2_ measurement fully disintegrates partly disintegrated samples, owing to the water motion caused by the magnetic stir bar. We could not afford full disintegration, because samples were analysed further for elemental stoichiometry (data reported elsewhere). Moreover, sample mass needs to be recorded after measurement and this is impossible after increased disintegration caused by stirring. The only way to record the mass of a sample before measurement without disturbing its physiology and microbiome is by buoyant mass, which is unreliable for water-saturated, disintegrated samples. This means that kelp laterals at an advanced stage of decomposition and, of course, those that had disintegrated entirely, are missing from our photosynthesis and respiration data. Given that warmer and dark treatments experienced faster decomposition in accordance with our predictions (data reported elsewhere), these data are missing non-randomly and difficult to impute. We assume missing data to represent cessation of photosynthesis, thereby conveying additional information that should be incorporated into inference. Data analysis was therefore split into naïve linear photosynthesis models that ignore missing data (i.e. complete case analysis), and therefore probably underestimate the treatment effect, and binomial models that incorporate the additional information contained in the unmeasurable disintegrated samples to model cessation of photosynthesis but necessarily binarize the response variable.

### Data analysis and visualization

Data analysis and visualization were performed in R v4.2.3 (R Core Team, 2023) with the tidyverse package family v2.0.0 (Wickham *et al.*, 2019) within the integrated development environment RStudio v2023.06.0+421 (RStudio Team, 2022). Vector files were edited and augmented in Affinity Designer v1.10.8 (Serif Ltd, Nottingham, UK). Hamiltonian Monte Carlo models were built with the *ulam* function of the rethinking package v2.21 (McElreath, 2019), an R interface to Stan (Carpenter *et al.*, 2017). All models were run with eight Markov chains spread across all cores, with 10^4^ iterations each, of which half were allocated to warm-up. Convergence and smooth sampling were ensured by assessing effective sample sizes and Rhat4 scores and visually scrutinizing trace and trace rank plots. All reported results are posterior probabilities and derived central tendencies and intervals. For detailed information on data analysis, see the Supplementary Data Methods.

In the first instance, a simple linear model with centred incubation time ($t-\bar{t}$, minutes) as the explanatory variable and dissolved O_2_ (micromolar) as the response variable (Supplementary Data Equation S1) was fit to the 30 measurements from each light and dark sample and blank incubation (see *Measurement of photosynthesis*). This yielded posterior probability distributions for slopes (micromolar per minute; Supplementary Data Fig. S1), which were converted to mass-based net and gross photosynthesis rates (micromoles per gram of dry mass per hour) and daily net photosynthesis rates (micromoles per gram of dry mass per day) (Supplementary Data Equation S3; Figs S2 and S3). Next, a multilevel multiple linear regression incorporating measurement error in the explanatory and response variables was fit to these rates as the response variable, using the standard deviations of slope posterior distributions as estimates of measurement error (Supplementary Data Equation S5). Primary explanatory variables were detrital age (days), treatment and tank (see *Decomposition experiment*). Partial pooling was applied to the tank variable to be able to make predictions for new tanks. Potential confounders included in the model were the initial incubation O_2_ (micromolar), mean incubation temperature (degrees Celsius), mean incubation pressure (hectopascals), incubation salinity (per mille) and sample dry mass (grams), of which only O_2_ turned out to show consistent enhancing effects on photosynthesis (Supplementary Data Figs S4 and S5B).

To complete the picture, we incorporated additional information contained in sample disintegration (see *Measurement of photosynthesis*) by calculating three binomial variables encoding different probabilistic aspects of detrital photosynthesis. Probability of autotrophy is defined as the probability that the detrital sample is autotrophic under saturating irradiance and therefore “1” was recorded for net photosynthesis greater than zero, and “0” was recorded for net photosynthesis less than or equal to zero. The probability of photosynthesis is defined as the probability that the detrital sample is photosynthetically viable under saturating irradiance, and therefore “1” was recorded for gross photosynthesis greater than zero, and “0” was recorded for gross photosynthesis equal to zero. Note that although theoretically gross photosynthesis is greater than or equal to zero, in practice measurement error can cause gross photosynthesis to be less than zero, in which case “0” was recorded too. The probability of daily autotrophy is a version of probability of autotrophy across a hypothetical 12-h-light-12-h-dark cycle with 12 h of saturating irradiance, and therefore “1” was recorded for daily net photosynthesis greater than zero, and “0” was recorded for daily net photosynthesis less than or equal to zero. In all cases, “0” was recorded if the sample was disintegrated. These new response variables were then modelled using a binomial generalized linear multilevel model with a logit link function and detrital age, treatment and tank as the explanatory variables (Supplementary Data Equation S6). Partial pooling was again applied to the tank variable to be able to make predictions for new tanks.

## RESULTS

**Fig. 2.**
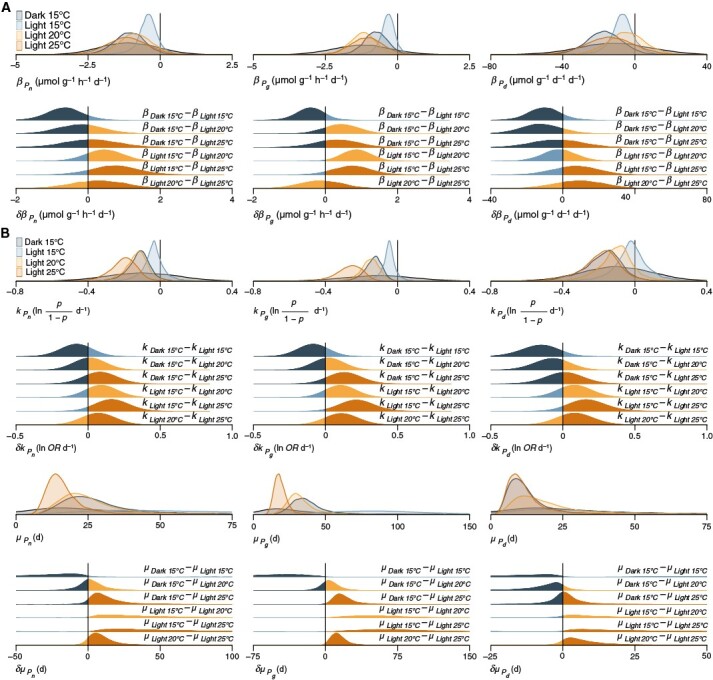
Probability distributions of relevant model parameters and their pairwise differences between treatments. Posterior probability distributions of *β*_Tr_ + *β*_Ta_, the slope of each treatment over detrital age including the variability across tanks (**A**, Supplementary Data Equation S5; **B**, Supplementary Data Equation S6) are coloured by treatment, and prior probability distributions for *β*_Tr_ are shown in black. In the case of the binomial generalized linear model with a logit link function (Supplementary Data Equation S6), *β*_Tr_ + *β*_Ta_ is termed *k*, because it corresponds to the logistic rate, i.e. log odds per day. The difference of log odds corresponds to the log odds ratio (*OR*). Derived prior and posterior quotient probability distributions of the sigmoid inflection point where *P* = 0.5 ( *μ* = $\frac{\alpha }{k}$) are also shown in **B**. For prior and posterior probability distributions of the intercept *ɑ* (Supplementary Data Equations S5 and S6), see Supplementary Data Figs S5A and S6.

**Fig. 3.**
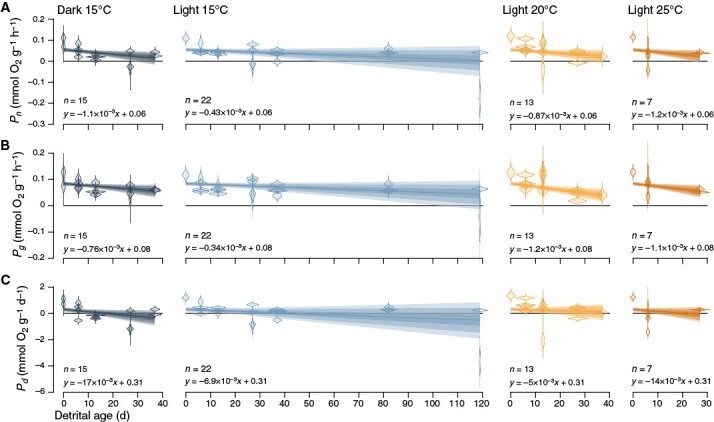
Effect of light and temperature on maximal (light-saturated) net (**A**), gross (**B**) and daily (24-h) net (**C**) detrital photosynthesis of *Ecklonia radiata*, given per gram of dry mass. Violins are posterior probability distributions for each of *n* observations, derived from *β*, the slope of O_2_ concentration over incubation time (Supplementary Data Equation S1) via conversion (Supplementary Data Equation S3). They give an indication of the measurement error that was incorporated into the final models as *s*_*P*_, the standard deviation of the posterior distribution of the slope (Supplementary Data Equation S5). Lines and intervals are means and 50, 80 and 90 % posterior probability intervals for *µ*, the mean prediction in relation to detrital age (Supplementary Data Equation S5). The intervals also incorporate variability between experimental tanks as *τ*, the standard deviation of the slope of photosynthesis over detrital age across tanks (Supplementary Data Equation S5). This means that given the priors and data, there is a 50, 80 and 90 % probability that the posterior prediction for a new experimental tank lies within the respective interval.

Our estimates of initial net (mean ± s.d.: 55 ± 6.1 µmol O_2_ g^−1^ dry mass h^−1^) and gross (84 ± 4.8 µmol O_2_ g^−1^ dry mass h^−1^) photosynthesis (Supplementary Data Fig. S5A) are very similar to prior data from our study site (Fig. 1D, see *Model species*). Over the course of our *ex situ* experiment, benthic *E. radiata* detritus photosynthesized up to our final sampling after 119 days. Given our data and priors, our first model (Supplementary Data Equation S5) predicts for new experimental tanks that net photosynthesis declines with detrital age at linear rates (*β*) of −1.1 ± 0.57 (*P*_*β* < 0_ = 98 %), −0.43 ± 0.41 (*P*_*β* < 0_ = 90 %), −0.87 ± 0.6 (*P*_*β* < 0_ = 93 %) and −1.2 ± 0.72 (*P*_*β* < 0_ = 95 %) µmol O_2_ g^−1^ dry mass h^−1^ day^−1^ in darkness, 15, 20 and 25 °C, respectively (Figs 2A and 3A). Although net photosynthesis clearly declines more slowly in light at 15 °C than darkness (*P*_*δβ* < 0_ = 92 %), 20 (*P*_*δβ* > 0_ = 81 %) and 25 °C (*P*_*δβ* > 0_ = 87 %), there is less of a difference between the three suboptimal treatments, with somewhat faster decline in darkness (*P*_*δβ* < 0_ = 63 %) and 25 °C (*P*_*δβ* > 0_ = 65 %) than at 20 °C (Fig. 2A). Hence, darkness and elevated temperature seem to have a similar diminishing effect on net detrital photosynthesis.

**Fig. 4.**
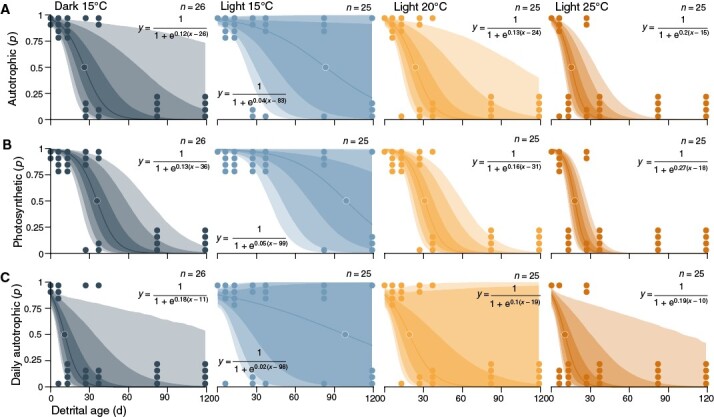
Effect of light and temperature on the probability of *Ecklonia radiata* detritus being autotrophic (**A**), photosynthetically active (**B**) and autotrophic over a 24-h period (**C**) under saturating irradiance. Lines and intervals are means and 50, 80 and 90 % posterior probability intervals for *µ*, the mean prediction in relation to detrital age (Supplementary Data Equation S6). The intervals also incorporate variability between experimental tanks as *τ*, the standard deviation of the slope of log odds over detrital age across tanks (Supplementary Data Equation S6). This means that given the priors and data, there is a 50, 80 and 90 % probability that the posterior prediction for a new experimental tank lies within the respective interval.

Model predictions suggest a different trend for gross photosynthesis, which declines at linear rates of −0.76 ± 0.45 (*P*_*β* < 0_ = 96 %), −0.34 ± 0.31 (*P*_*β* < 0_ = 91 %), −1.2 ± 0.46 (*P*_*β* < 0_ = 99 %) and −1.1 ± 0.58 (*P*_*β* < 0_ = 97 %) µmol O_2_ g^−1^ dry mass h^−1^ day^−1^ in darkness, 15, 20 and 25 °C, respectively (Figs 2A and 3B). Although gross photosynthesis also declines more slowly in light at 15 °C than in darkness (*P*_*δβ* < 0_ = 88 %), 20 (*P*_*δβ* > 0_ = 98 %) and 25 °C (*P*_*δβ* > 0_ = 92 %), darkness causes an appreciably slower decline in gross photosynthesis than 20 (*P*_*δβ* > 0_ = 81 %) and 25 °C (*P*_*δβ* > 0_ = 70 %) (Fig. 2A). This indicates that gross detrital photosynthesis is affected by temperature more than by darkness, and this difference disappears in net detrital photosynthesis because of elevated respiration. Hence, the observed temperature effect is primarily caused by declines in photosynthesis (*β*_*P*n_ ≈ *β*_*P*g_), whereas the effect of darkness seems to be caused by elevated respiration (*β*_*P*n_ ≠ *β*_*P*g_).

Predictions for daily net photosynthesis further clarify the observed differences between net photosynthesis and gross photosynthesis. Daily net photosynthesis declines at linear rates of −17 ± 11 (*P*_*β* < 0_ = 95 %), −6.9 ± 7.9 (*P*_*β* < 0_ = 87 %), −5 ± 11 (*P*_*β* < 0_ = 68 %) and −14 ± 14 (*P*_*β* < 0_ = 85 %) µmol O_2_ g^−1^ dry mass day^−1^ day^−1^ in darkness, 15, 20 and 25 °C, respectively (Figs 2A and 3C). Here, the dark treatment stands out, with a faster decline in daily net photosynthesis than 15 (*P*_*δβ* < 0_ = 88 %), 20 (*P*_*δβ* < 0_ = 86 %) and 25 °C (*P*_*δβ* < 0_ = 59 %) (Fig. 2A). This supports the idea that darkness primarily causes an increase in detrital respiration, whereas elevated temperature primarily diminishes detrital photosynthesis.

To incorporate cases where measurements of photosynthesis were impossible to obtain but complete decomposition is known, and thus to make use of all the available information and alleviate the bias caused by non-random data missingness, detrital photosynthesis was also explored from a probabilistic standpoint (Supplementary Data Equation S6). According to this second model, given our data and priors, there is near certainty of autotrophy (log odds = 3.1 ± 0.64, *P* = 96 %) and photosynthesis (log odds = 4.8 ± 0.93, *P* = 99 %) at the start of the experiment (Supplementary Data Fig. S6). Probability of autotrophy in new experimental tanks then declines at logistic rates (*k*) of −0.12 ± 0.07 (*P*_*k* < 0_ = 97 %), −0.04 ± 0.06 (*P*_*k* < 0_ = 78 %), −0.13 ± 0.07 (*P*_*k* < 0_ = 98 %) and −0.2 ± 0.08 (*P*_*k* < 0_ = 99 %) log odds day^−1^ in darkness, 15, 20 and 25 °C, respectively (Figs 2B and 4A). The light 15 °C treatment has slower logistic decay compared with darkness (*P*_*δk* < 0_ = 97 %), 20 (*P*_*δk* > 0_ = 98 %) and 25 °C (*P*_*δk* > 0_ = 100 %) (Fig. 2B). Homologous to net photosynthesis (Figs 2A and 3A), the dark and 20 °C treatments have similar effects on the probability of autotrophy (*P*_*δk* > 0_ = 60 %), but in contrast, 25 °C has a distinctly stronger negative effect than 20 °C (*P*_*δk* > 0_ = 89 %) (Fig. 2B). This highlights the effect of incorporating cases of complete decomposition, which were most prevalent in the 25 °C treatment. Correspondingly, the number of days after which *P* = 0.5 (*µ*) occurs 57, 59 and 68 days later in light at 15 °C than in darkness (*P*_*δµ* < 0_ = 78 %), 20 (*P*_*δµ* > 0_ = 79 %) and 25 °C (*P*_*δµ* > 0_ = 79 %), respectively (Figs 2B and 4A).

As with gross photosynthesis, model predictions suggest a different trend for the probability that detritus is photosynthetically active, which declines at logistic rates of −0.13 ± 0.06 (*P*_*k* < 0_ = 99 %), −0.05 ± 0.05 (*P*_*k* < 0_ = 91 %), −0.16 ± 0.06 (*P*_*k* < 0_ = 99 %) and −0.27 ± 0.08 (*P*_*k* < 0_ = 100 %) log odds day^−1^ in darkness, 15, 20 and 25 °C, respectively (Figs 2B and 4B). The light 15 °C treatment has slower logistic decay than darkness (*P*_*δk* < 0_ = 99 %), 20 (*P*_*δk* > 0_ = 99 %) and 25 °C (*P*_*δk* > 0_ = 100 %) (Fig. 2B). In contrast to gross photosynthesis (Figs 2A and 3B), the dark and 20 °C treatments have more similar (*P*_*δk* > 0_ = 69 %) effects, and 25 °C has a stronger negative effect than 20 °C (*P*_*δk* > 0_ = 98 %). Correspondingly, halving of probability takes 63, 68 and 81 days longer in light at 15 °C than in darkness (*P*_*δµ* < 0_ = 91 %), 20 (*P*_*δµ* > 0_ = 91 %) and 25 °C (*P*_*δµ* > 0_ = 91 %), respectively (Figs 2B and 4B). Thus, the probability of autotrophy and photosynthesis are more similar than net and gross photosynthesis, and the putative effect of respiration seems to be somewhat alleviated here by data binarization.

The probability of daily autotrophy declines at logistic rates of −0.18 ± 0.12 (*P*_*k* < 0_ = 96 %), −0.02 ± 0.11 (*P*_*k* < 0_ = 61 %), −0.1 ± 0.11 (*P*_*k* < 0_ = 88 %) and −0.19 ± 0.12 (*P*_*k* < 0_ = 96 %) log odds day^−1^ in darkness, 15, 20 and 25 °C, respectively (Figs 2B and 4C). The light 15 °C treatment has slower logistic decay compared with darkness (*P*_*δk* < 0_ = 99 %), 20 (*P*_*δk* > 0_ = 91 %) and 25 °C (*P*_*δk* > 0_ = 99 %) (Fig. 2B). Homologous to daily net photosynthesis (Figs 2A and 3C), darkness causes a faster decline than 20 °C (*P*_*δk* < 0_ = 86 %), but conversely, 25 °C tends towards a stronger negative effect (*P*_*δk* > 0_ = 56 %) (Fig. 2B). Correspondingly, the inflection point of the sigmoid is reached 87, 79 and 88 days later in light at 15 °C than in darkness (*P*_*δµ* < 0_ = 64 %), 20 (*P*_*δµ* > 0_ = 64 %) and 25 °C (*P*_*δµ* > 0_ = 64 %), respectively (Figs 2B and 4C). The effect of elevated detrital respiration in darkness therefore becomes evident only when exploring binary data on a 24-h basis.

In summary, elevated temperature of >15 °C reduces the potential for *E. radiata* detritus to remain photosynthetically viable, as evidenced by two separate types of models (Figs 2–4; Supplementary Data Equations S5 and S6). *T*_opt_ for detrital photosynthesis is therefore ≤15 °C. Furthermore, detritus remains viable for longer under subcompensating irradiance (*E*_max_ = 8 µmol photons m^−2^ s^−1^) compared with complete darkness (Figs 2–4). The main difference between the negative effects of elevated temperature and reduced light on detrital photosynthesis is that the former seems to primarily decrease photosynthesis whereas the latter primarily increases respiration. At 15 °C and *E*_max_ = 8 µmol photons m^−2^ s^−1^, the optimal conditions within the constraints of our experiment, detrital photosynthesis persists for ≥119 days. Finally, both modelling strategies (Figs 2–4) tell similar stories to some extent, but the probabilistic view (Figs 2B and 4) revealed underestimation of the effect of temperature, especially between 20 and 25 °C, as a major artefact of non-random data missingness.

## DISCUSSION

We provide evidence for elevated temperature sensitivity of detritus relative to intact kelp. Our data suggest that detrital photosynthesis is a mechanism underlying the accelerating effect of temperature on kelp carbon remineralization (Litchfield *et al.*, 2020; Frontier *et al.*, 2021*a*; Pedersen *et al.*, 2021; Filbee-Dexter *et al.*, 2022). We also show continued detrital photosynthesis below compensating irradiance, implying that it is a meaningful mechanism below depths inhabited by the attached alga. Finally, our study provides new evidence for photosynthetically viable kelp detritus in the Indian Ocean, surpassing the only previous reports for both *ex situ* (Frontier *et al.*, 2021*b*) and *in situ* (Wright *et al.*, 2022) persistence of detrital O_2_ production by 63 and 87 days, respectively.

The mechanism underlying the thermal driver of remineralization might have been partly misattributed. Heterotrophic activity is usually considered to be the main mechanism behind the temperature effect (Litchfield *et al.*, 2020; Frontier *et al.*, 2021*a*; Pedersen *et al.*, 2021; Filbee-Dexter *et al.*, 2022), owing to the temperature dependence of heterotrophic metabolism (Yvon-Durocher *et al.*, 2012; Wilken *et al.*, 2013; Smale *et al.*, 2017). We speculated that intrinsic mechanisms could also play a role, and detrital photosynthesis turns out to be an example of this. Given that detrital photosynthesis can decelerate (Birch *et al.*, 1983; Brouwer, 1996; de Bettignies *et al.*, 2020) or even counterbalance and reverse (Frontier *et al.*, 2021*a*, *b*; Pedersen *et al.*, 2021; Filbee-Dexter *et al.*, 2022; Wright *et al.*, 2022) decomposition, accelerated decline of detrital photosynthesis with increasing temperature (Figs 2–4) might, at least in part, drive faster remineralization at elevated temperatures (Litchfield *et al.*, 2020; Frontier *et al.*, 2021*a*; Pedersen *et al.*, 2021; Filbee-Dexter *et al.*, 2022). This finding highlights that intrinsic mechanisms related to detrital physiology might also need to be considered. By comparing the temperature response of net and gross O_2_ production, we show that temperature primarily affects detrital photosynthesis rather than detrital respiration, and net and gross photosynthesis therefore seem to have similar *T*_opt_ (Figs 2–4). This finding differs from the short-term thermal response of kelp, where respiration primarily increases with temperature, causing *T*_opt_ of gross photosynthesis to be higher than that of net photosynthesis (Fig. 1D; Davison *et al.*, 1991; Staehr and Wernberg, 2009; Andersen *et al.*, 2013; Terada *et al.*, 2016; Wernberg *et al.*, 2016). This difference might be partly explained by a lower metabolic capacity of detritus, and the combination of detrital and heterotrophic microbial respiration, which presumably respond inversely to detrital age. Consequently, our observed negative effect of temperature on photosynthesis might be either a direct negative effect of temperature on the photosynthetic apparatus caused by long-term exposure or an indirect effect of increased microbial and/or meiofaunal heterotrophy (macrofauna were excluded in our experiment), which would make detrital photosynthesis a secondary mechanism or feedback (*sensu*Wright *et al.*, 2022). More microbiological research is required to tease apart the sequence of cause and effect of these mechanisms.

Detrital temperature and light tolerances are substantially different from those of the intact plant. Based on our meta-analysis (Fig. 1B–D), we predicted that *T*_opt_ and *E*_c_ for *E. radiata* detritus would lie at or above 18 °C and 13 µmol photons m^−2^ s^−1^, respectively, but neither prediction was supported. The *T*_opt_ of detritus is in fact ≥3 °C colder than our prediction for intact kelp, lying at or below our lowest experimental temperature (Figs 2–4). More specifically, *T*_opt_ for net (Figs 2A and 3A) and gross (Figs 2A and 3B) detrital photosynthesis are at least 9 and 10 °C colder, respectively, than those derived from net and gross sporophyte photosynthesis at our study site after short-term temperature exposure (Fig. 1D; Staehr and Wernberg, 2009; Wernberg *et al.*, 2016). This finding is supported by reduced *T*_opt_ with increasing duration of temperature exposure for whole but decomposing brown macroalgae, including *E. radiata* (Xiao *et al.*, 2015). Given that *E. radiata* is one of the most thermotolerant kelps (Fig. 1A, B), its colder detrital *T*_opt_ suggests that photosynthesis is generally more thermosensitive in the detrital phase. Considering the clear evidence of detrital photosynthesis in our experiment, *E*_c_ of detritus is ≥5 µmol photons m^−2^ s^−1^ below our prediction for intact kelp. Interestingly, detrital photosynthesis declined faster in darkness than subcompensating irradiance (Figs 2–4). This result implies that detrital photosynthesis is a mechanism underlying reduced light as a driver of remineralization (Frontier *et al.*, 2021*a*, *b*; Pedersen *et al.*, 2021) and persists longer in light than in darkness regardless of the amount of irradiance. Within the limits of our experiment, these findings mean that the lower the temperature and the higher the irradiance, the better for persistence of detrital photosynthetic viability. *Ecklonia radiata* is known to have a high capacity for photoacclimation (Miller *et al.*, 2006), and the observed extraordinary level of persistence under subcompensating light might be attributable to this predisposition. However, there are several kelps adapted to similar (Davison *et al.*, 1991; Andersen *et al.*, 2013; Terada *et al.*, 2016) or even dimmer (García-Sánchez *et al.*, 2016) light regimes, potentially extending our inference to kelps at large and suggesting that most kelp detritus in the photic zone is photosynthetically viable.

Our findings have important implications for kelp carbon cycling. Ocean warming caused by anthropogenic climate change might be altering the kelp carbon cycle directly, by accelerating decomposition (Filbee-Dexter *et al.*, 2022), and indirectly, by causing poleward range shifts that favour species with more labile detritus in temperate regions (Wright *et al.*, 2022), potentially creating vicious circles. Our findings suggest that detrital photosynthesis, a mechanism underlying the direct effect of ocean warming on remineralization, is more thermosensitive than expected. Increases in temperature, no matter what magnitude, will cause a decline in detrital photosynthesis and, consequently, detrital longevity. But deeper waterbodies with temperatures closer to the colder thermal optimum of detritus might still receive enough light to allow persistence of photosynthetic viability in the detrital phase. These deeper waters are usually associated with the most promising sedimentary and ocean carbon sinks (Krause-Jensen and Duarte, 2016; Pessarrodona *et al.*, 2023). Our findings on the difference between darkness and subcompensating light indicate that detrital photosynthesis might even play a role in the mesophotic zone. This means that exported kelp detritus might remain photosynthetically viable at depths well below the kelp forest and thus resist or delay decomposition. Research on deep-water kelp photosynthesis and detrital production (Dominik and Zimmerman, 2006; Marins *et al.*, 2014; García-Sánchez *et al.*, 2016) is especially exciting in this context. Mesophotic kelp forests thrive at light levels as low as ~1.4 µmol photons m^−2^ s^−1^ (Marins *et al.*, 2014; Buglass *et al.*, 2022) and are closer to potential carbon sinks. If these kelps exhibit detrital photosynthesis, their detritus would not only be photosynthetically viable in the mesophotic zone, like that of *E. radiata*, but actively photosynthesizing in the proximity of deep carbon sinks.

In conclusion, we show that photosynthesis in the detrital phase is (1) an important primary or secondary mechanism underlying the temperature and light dependence of decomposition, (2) less tolerant to elevated temperature and more tolerant to low light when compared to photosynthesis in the intact plant and (3) remains possible after ≥119 days post-excision under subcompensating irradiance. These inferences translate to greater sensitivity of detritus to ocean warming relative to intact kelp, in addition to potential maintenance of detrital carbon assimilation below the photoenvironment inhabited by the attached alga and, consequently, closer to deep carbon sinks. Greater detrital photosynthetic viability at or below 15 °C in the photic zone suggests the possibility of extended carbon transport following export and, subsequently, higher carbon sequestration potential if these conditions are met.

## SUPPLEMENTARY DATA

Supplementary data are available at *Annals of Botany* online and consist of the following.

Figure S1: Dissolved O_2_ model parameters. Figure S2: Blank correction of slopes. Figure S3: Calculation of gross photosynthesis. Figure S4: Effect of confounding variables associated with incubation. Figure S5: Additional linear photosynthesis model parameters. Figure S6: Additional binomial photosynthesis model parameters.

mcad167_suppl_Supplementary_Material

## Data Availability

Data and annotated code are available at github.com/lukaseamus/detrital-tolerance. We place no restrictions on data and code availability within the constraints of the specified copyleft licence: GNU General Public License.

## References

[CIT0001] Andersen GS , Pedersen MF, Nielsen SL. 2013. Temperature acclimation and heat tolerance of photosynthesis in Norwegian *Saccharina latissima* (Laminariales, Phaeophyceae). Journal of Phycology 49: 689–700. 27007201 10.1111/jpy.12077

[CIT0002] Arnosti C. 2011. Microbial extracellular enzymes and the marine carbon cycle. Annual Review of Marine Science 3: 401–425. 10.1146/annurev-marine-120709-142731 21329211

[CIT0003] de Bettignies F , Dauby P, Thomas F, et al. 2020. Degradation dynamics and processes associated with the accumulation of *Laminaria hyperborea* (Phaeophyceae) kelp fragments: an in situ experimental approach. Journal of Phycology 56: 1481–1492. 32557584 10.1111/jpy.13041

[CIT0004] Birch PB , Gabrielson JO, Hamel KS. 1983. Decomposition of *Cladophora*. I. Field studies in the Peel-Harvey estuarine system, Western Australia. Botanica Marina 26: 165–172.

[CIT0005] Blain CO , Shears NT. 2019. Seasonal and spatial variation in photosynthetic response of the kelp *Ecklonia radiata* across a turbidity gradient. Photosynthesis Research 140: 21–38. 30877516 10.1007/s11120-019-00636-7

[CIT0006] Blain CO , Shears NT. 2020. Nutrient enrichment offsets the effects of low light on growth of the kelp *Ecklonia radiata*. Limnology and Oceanography 65: 2220–2235.

[CIT0007] Blain CO , Rees TAV, Hansen SC, Shears NT. 2020. Morphology and photosynthetic response of the kelp *Ecklonia radiata* across a turbidity gradient. Limnology and Oceanography 65: 529–544.

[CIT0008] Blain CO , Hansen SC, Shears NT. 2021. Coastal darkening substantially limits the contribution of kelp to coastal carbon cycles. Global Change Biology 27: 5547–5563. 34382288 10.1111/gcb.15837

[CIT0009] Brouwer PEM. 1996. Decomposition in situ of the sublittoral Antarctic macroalga *Desmarestia anceps* Montagne. Polar Biology 16: 129–137.

[CIT0010] Buglass S , Kawai H, Hanyuda T, et al. 2022. Novel mesophotic kelp forests in the Galápagos archipelago. Marine Biology 169: 156.

[CIT0011] Carpenter B , Gelman A, Hoffman MD, et al. 2017. Stan: a probabilistic programming language. Journal of Statistical Software 76: 1–32. 36568334 10.18637/jss.v076.i01 PMC9788645

[CIT0012] Cebrián J , Lartigue J. 2004. Patterns of herbivory and decomposition in aquatic and terrestrial ecosystems. Ecological Monographs 74: 237–259.

[CIT0013] Davison IR , Greene RM, Podolak EJ. 1991. Temperature acclimation of respiration and photosynthesis in the brown alga *Laminaria saccharina*. Marine Biology 110: 449–454.

[CIT0014] Dominik CM , Zimmerman RC. 2006. Dynamics of carbon allocation in a deep-water population of the deciduous kelp *Pleurophycus gardneri* (Laminariales). Marine Ecology Progress Series 309: 143–157.

[CIT0015] Duarte CM , Gattuso J-P, Hancke K, et al. 2022. Global estimates of the extent and production of macroalgal forests. Global Ecology and Biogeography 31: 1422–1439.

[CIT0016] Enríquez S , Duarte CM, Sand-Jensen K. 1993. Patterns in decomposition rates among photosynthetic organisms: the importance of detritus C:N:P content. Oecologia 94: 457–471. 28313985 10.1007/BF00566960

[CIT0017] Fairhead VA , Cheshire AC. 2004. Seasonal and depth related variation in the photosynthesis–irradiance response of *Ecklonia radiata* (Phaeophyta, Laminariales) at West Island, South Australia. Marine Biology 145: 415–426.

[CIT0018] Filbee-Dexter K , Feehan CJ, Smale DA, et al. 2022. Kelp carbon sink potential decreases with warming due to accelerating decomposition. PLoS Biology 20: e3001702. 35925899 10.1371/journal.pbio.3001702 PMC9352061

[CIT0019] Fraser CI , Morrison AK, Hogg AM, et al. 2018. Antarctica’s ecological isolation will be broken by storm-driven dispersal and warming. Nature Climate Change 8: 704–708.

[CIT0020] Frontier N , de Bettignies F, Foggo A, Davoult D. 2021b. Sustained productivity and respiration of degrading kelp detritus in the shallow benthos: detached or broken, but not dead. Marine Environmental Research 166: 105277. 33592375 10.1016/j.marenvres.2021.105277

[CIT0021] Frontier N , Mulas M, Foggo A, Smale DA. 2021a. The influence of light and temperature on detritus degradation rates for kelp species with contrasting thermal affinities. Marine Environmental Research 173: 105529. 34800869 10.1016/j.marenvres.2021.105529

[CIT0022] García-Sánchez MJ , Delgado-Huertas A, Fernández JA, Flores-Moya A. 2016. Photosynthetic use of inorganic carbon in deep-water kelps from the Strait of Gibraltar. Photosynthesis Research 127: 295–305. 26275764 10.1007/s11120-015-0184-z

[CIT0023] GBIF. 2023. *Ecklonia radiata* occurrence download. 10.15468/dl.wpf65h. 22 October 2023.

[CIT0024] Graham MH , Kinlan BP, Druehl LD, Garske LE, Banks S. 2007. Deep-water kelp refugia as potential hotspots of tropical marine diversity and productivity. Proceedings of the National Academy of Sciences of the United States of America 104: 16576–16580. 17913882 10.1073/pnas.0704778104 PMC2034254

[CIT0025] Krause-Jensen D , Duarte CM. 2016. Substantial role of macroalgae in marine carbon sequestration. Nature Geoscience 9: 737–742.

[CIT0026] Küpper FC , Müller DG, Peters AF, Kloareg B, Potin P. 2002. Oligoalginate recognition and oxidative burst play a key role in natural and induced resistance of sporophytes of Laminariales. Journal of Chemical Ecology 28: 2057–2081. 12474900 10.1023/a:1020706129624

[CIT0027] Litchfield SG , Schulz KG, Kelaher BP. 2020. The influence of plastic pollution and ocean change on detrital decomposition. Marine Pollution Bulletin 158: 111354. 32753168 10.1016/j.marpolbul.2020.111354

[CIT0028] Macaya EC , Boltana S, Hinojosa IA, et al. 2005. Presence of sporophylls in floating kelp rafts of *Macrocystis* spp. (Phaeophyceae) along the Chilean Pacific coast. Journal of Phycology 41: 913–922.

[CIT0029] Marins BV , Amado-Filho GM, Barbarino E, Pereira-Filho GH, Longo LL. 2014. Seasonal changes in population structure of the tropical deep-water kelp *Laminaria abyssalis*. Phycological Research 62: 55–62.

[CIT0030] Marzinelli EM , Williams SB, Babcock RC, et al. 2015. Large-scale geographic variation in distribution and abundance of Australian deep-water kelp forests. PLoS One 10: e0118390. 25693066 10.1371/journal.pone.0118390 PMC4334971

[CIT0031] McElreath R. 2019. *Statistical rethinking*^*2*^: *a Bayesian course with examples in R and Stan*. 2nd edn. Boca Raton: Chapman & Hall/CRC.

[CIT0032] Medlyn BE , Loustau D, Delzon S. 2002. Temperature response of parameters of a biochemically based model of photosynthesis. I. Seasonal changes in mature maritime pine (*Pinus pinaster* Ait). Plant, Cell & Environment 25: 1155–1165.

[CIT0033] Miller HL III , Dunton KH. 2007. Stable isotope (^13^C) and O_2_ micro-optode alternatives for measuring photosythesis in seaweeds. Marine Ecology Progress Series 329: 85–97.

[CIT0034] Miller SM , Wing SR, Hurd CL. 2006. Photoacclimation of *Ecklonia radiata* (Laminariales, Heterokontophyta) in Doubtful Sound, Fjordland, Southern New Zealand. Phycologia 45: 44–52.

[CIT0035] OBIS. 2023. *Ecklonia radiata* occurrence download. https://obis.org/taxon/214344. 8 May 2023.

[CIT0036] Pedersen MF , Filbee-Dexter K, Frisk NL, Sárossy Z, Wernberg T. 2021. Carbon sequestration potential increased by incomplete anaerobic decomposition of kelp detritus. Marine Ecology Progress Series 660: 53–67.

[CIT0037] Pessarrodona A , Assis J, Filbee-Dexter K, et al. 2022b. Global seaweed productivity. Science Advances 8: eabn2465. 36103524 10.1126/sciadv.abn2465 PMC9473579

[CIT0038] Pessarrodona A , Filbee-Dexter K, Krumhansl KA, Pedersen MF, Moore PJ, Wernberg T. 2022a. A global dataset of seaweed net primary productivity. Scientific Data 9: 484. 35933515 10.1038/s41597-022-01554-5 PMC9357081

[CIT0039] Pessarrodona A , Franco-Santos RM, Wright LS, et al. 2023. Carbon sequestration and climate change mitigation using macroalgae: a state of knowledge review. Biological Reviews of the Cambridge Philosophical Society 98: 1945–1971. 37437379 10.1111/brv.12990

[CIT0040] Queirós AM , Stephens N, Widdicombe S, et al. 2019. Connected macroalgal‐sediment systems: blue carbon and food webs in the deep coastal ocean. Ecological Monographs 89: e01366.

[CIT0041] R Core Team. 2023. R: a language and environment for statistical computing. Vienna: R Foundation for Statistical Computing.

[CIT0042] Randall J , Wotherspoon S, Ross J, Hermand J-P, Johnson CR. 2019. An *in situ* study of production from diel oxygen modelling, oxygen exchange, and electron transport rate in the kelp *Ecklonia radiata*. Marine Ecology Progress Series 615: 51–65.

[CIT0043] Ravaglioli C , Bulleri F, Rühl S, et al. 2019. Ocean acidification and hypoxia alter organic carbon fluxes in marine soft sediments. Global Change Biology 25: 4165–4178. 31535452 10.1111/gcb.14806

[CIT0044] Rodgers KL , Shears NT. 2016. Modelling kelp forest primary production using in situ photosynthesis, biomass and light measurements. Marine Ecology Progress Series 553: 67–79.

[CIT0045] Rodgers KL , Rees TAV, Shears NT. 2015. A novel system for measuring *in situ* rates of photosynthesis and respiration of kelp. Marine Ecology Progress Series 528: 101–115.

[CIT0046] Rothäusler E , Gómez I, Hinojosa IA, et al. 2011. Kelp rafts in the Humboldt Current: interplay of abiotic and biotic factors limit their floating persistence and dispersal potential. Limnology and Oceanography 56: 1751–1763.

[CIT0047] Rothäusler E , Reinwald H, López BA, Tala F, Thiel M. 2018. High acclimation potential in floating *Macrocystis pyrifera* to abiotic conditions even under grazing pressure – a field study. Journal of Phycology 54: 368–379. 29533462 10.1111/jpy.12643

[CIT0048] RStudio Team. 2022. RStudio: integrated development environment for R. Boston: RStudio, Inc.

[CIT0049] Smale DA , Taylor JD, Coombs SH, Moore G, Cunliffe M. 2017. Community responses to seawater warming are conserved across diverse biological groupings and taxonomic resolutions. Proceedings of the Royal Society B: Biological Sciences 284: 20170534. 10.1098/rspb.2017.0534 PMC5597821 28878056

[CIT0050] Sosik EA , Simenstad CA. 2013. Isotopic evidence and consequences of the role of microbes in macroalgae detritus-based food webs. Marine Ecology Progress Series 494: 107–119.

[CIT0051] Staehr PA , Wernberg T. 2009. Physiological responses of *Ecklonia radiata* (Laminariales) to a latitudinal gradient in ocean temperature. Journal of Phycology 45: 91–99. 27033648 10.1111/j.1529-8817.2008.00635.x

[CIT0052] Tala F , Gómez I, Luna-Jorquera G, Thiel M. 2013. Morphological, physiological and reproductive conditions of rafting bull kelp (*Durvillaea antarctica*) in northern-central Chile (30°S). Marine Biology 160: 1339–1351.

[CIT0053] Tala F , López BA, Velásquez M, et al. 2019. Long-term persistence of the floating bull kelp *Durvillaea antarctica* from the South-East Pacific: potential contribution to local and transoceanic connectivity. Marine Environmental Research 149: 67–79. 31154063 10.1016/j.marenvres.2019.05.013

[CIT0054] Terada R , Shikada S, Watanabe Y, et al. 2016. Effect of PAR and temperature on the photosynthesis of the Japanese alga, *Ecklonia radicosa* (Laminariales), based on field and laboratory measurements. Phycologia 55: 178–186.

[CIT0055] Trevathan-Tackett SM , Kelleway J, Macreadie PI, Beardall J, Ralph P, Bellgrove A. 2015. Comparison of marine macrophytes for their contributions to blue carbon sequestration. Ecology 96: 3043–3057. 27070023 10.1890/15-0149.1

[CIT0056] Veenhof RJ , Champion C, Dworjanyn SA, Schwoerbel J, Visch W, Coleman MA. 2023. Projecting kelp (*Ecklonia radiata*) gametophyte thermal adaptation and persistence under climate change. Annals of Botany 133: 153–167. 10.1093/aob/mcad132 PMC10921825 37665952

[CIT0057] Wernberg T , de Bettignies T, Joy BA, Finnegan PM. 2016. Physiological responses of habitat‐forming seaweeds to increasing temperatures. Limnology and Oceanography 61: 2180–2190.

[CIT0058] Wickham H , Averick M, Bryan J, et al. 2019. Welcome to the Tidyverse. Journal of Open Source Software 4: 1686.

[CIT0059] Wilken S , Huisman J, Naus-Wiezer S, Van Donk E. 2013. Mixotrophic organisms become more heterotrophic with rising temperature. Ecology Letters 16: 225–233. 23173644 10.1111/ele.12033

[CIT0060] Wright LS. Kelp temperature tolerance. 2023. Zenodo v1.0.1: (22 October 2023, Date of deposit; 7 December 2023, Date accessed). doi:10.5281/zenodo.10031207

[CIT0061] Wright LS , Foggo A. 2021. Photosynthetic pigments of co-occurring Northeast Atlantic *Laminaria* spp. are unaffected by decomposition. Marine Ecology Progress Series 678: 227–232.

[CIT0062] Wright LS , Kregting L. 2023. Genus-specific response of kelp photosynthetic pigments to decomposition. Marine Biology 170: 144.

[CIT0063] Wright LS , Pessarrodona A, Foggo A. 2022. Climate-driven shifts in kelp forest composition reduce carbon sequestration potential. Global Change Biology 28: 5514–5531. 35694894 10.1111/gcb.16299 PMC9545355

[CIT0064] Xiao X , de Bettignies T, Olsen YS, Agusti S, Duarte CM, Wernberg T. 2015. Sensitivity and acclimation of three canopy-forming seaweeds to UVB radiation and warming. PLoS One 10: e0143031. 26630025 10.1371/journal.pone.0143031 PMC4668109

[CIT0065] Yvon-Durocher G , Caffrey JM, Cescatti A, et al. 2012. Reconciling the temperature dependence of respiration across timescales and ecosystem types. Nature 487: 472–476. 22722862 10.1038/nature11205

