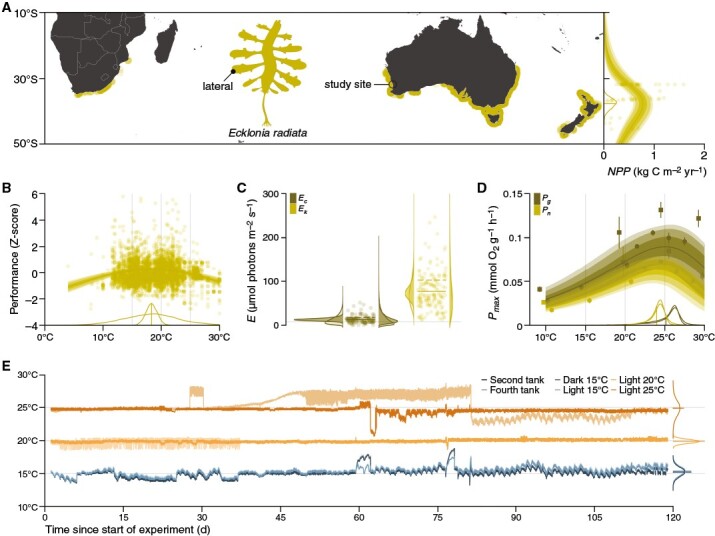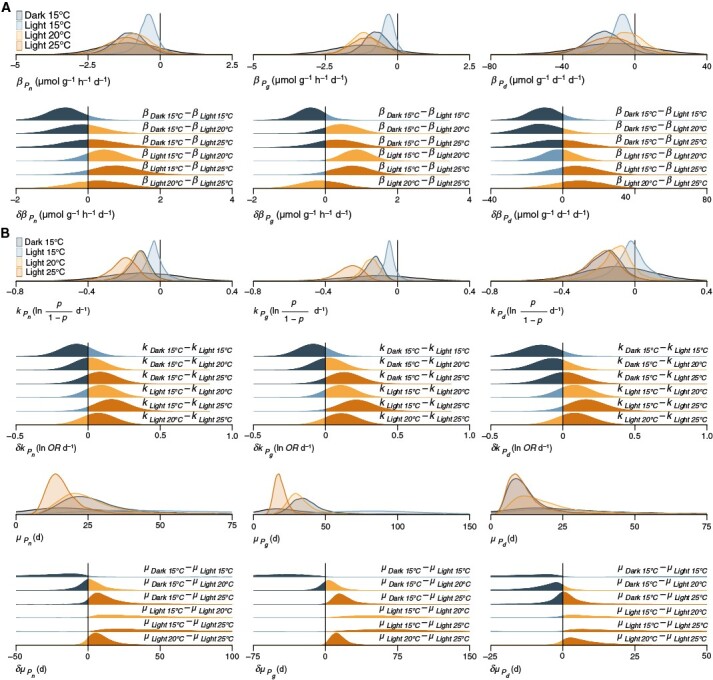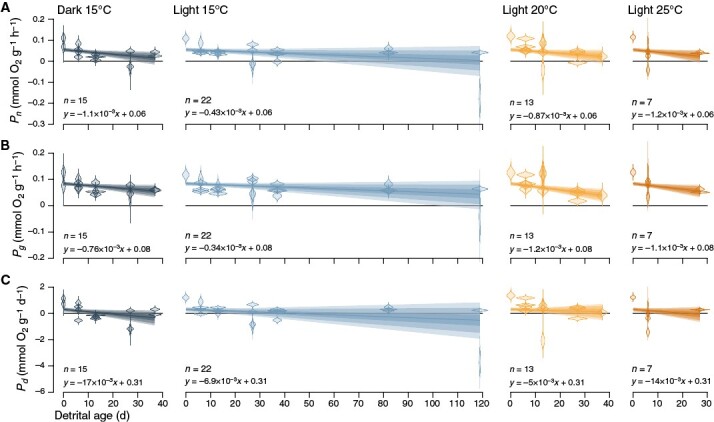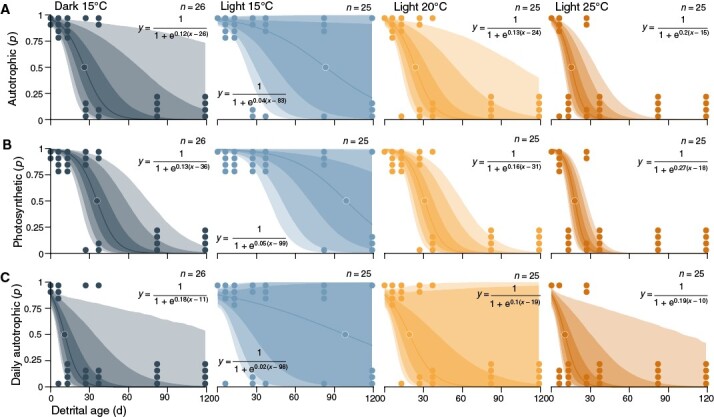# Correction to: Temperature sensitivity of detrital photosynthesis

**DOI:** 10.1093/aob/mcae013

**Published:** 2024-02-18

This is a correction to: Luka Seamus Wright, Taylor Simpkins, Karen Filbee-Dexter, Thomas Wernberg, Temperature sensitivity of detrital photosynthesis, *Annals of Botany*, 2023; mcad167, https://doi.org/10.1093/aob/mcad167.

The originally published version of this manuscript contained a number of erroneous in-text figure references. These have been corrected.

Additionally, in Panel A of Figure 2, the two equations in the final column contained a typographic error. These read, respectively, $\beta_{P_{d}}(\mu mol\;g^{-1}\;h^{-1}\;d^{-1})$ and $\delta \beta_{P_{d}}(\mu mol\;g^{-1}\;h^{-1}\;d^{-1})$. These have been corrected to, respectively, $\beta_{P_{d}}(\mu mol\;g^{-1}\;d^{-1}\;d^{-1})$ and $\delta \beta_{P_{d}}(\mu mol\;g^{-1}\;d^{-1}\;d^{-1})$. Furthermore, in the equation in the penultimate sentence of the legend to Figure 2, the letter ‘k’ was changed from upper case to lower case.

The publisher apologizes for these errors and would like to make clear that they were not the fault of the authors.