# Supplementary material for: Temperature sensitivity of detrital photosynthesis
Source: Ann Bot. 2023 Dec 24;133(1):17–28. doi: 10.1093/aob/mcad167 (PMC10921823; doi:10.1093/aob/mcad167)
Supplement: mcad167_suppl_Supplementary_Material [file mcad167_suppl_supplementary_material.pdf]

Supplement to
Temperature sensitivity of detrital photosynthesis

Luka Seamus Wright[1,2]*, Taylor Simpkins[1,2], Karen Filbee-Dexter[1,2,3] and Thomas Wernberg[1,2,3]
[1]Oceans Institute, University of Western Australia, Perth, Australia
[2]School of Biological Sciences, University of Western Australia, Perth, Australia
[3]Institute of Marine Research, His, Norway
*Corresponding author: luka@wright.it, luka.wright@research.uwa.edu.au

Supplementary methods: data analysis
In the first instance, dissolved O_2 (O, µM) in light and dark sample and blank incubations was modelled as a function of centred incubation time ($t_c = t - \bar{t}$, min) as

$$O \sim \text{normal}(\text{mean} = \mu, \text{standard deviation} = \sigma) \quad (1)$$
$$\mu = \alpha + \beta \times t_c$$
$$\alpha \sim \text{normal}(\text{mean} = 228, \text{standard deviation} = 20)$$
$$\beta \sim \text{normal}(\text{mean} = 1.4, -0.69, 0, \text{standard deviation} = 1, 0.5, 0.5)$$
$$\sigma \sim \text{exponential}(\text{rate} = 1)$$

where the first line is the Gaussian likelihood function describing the response variable, the second is the linear model describing μ, and the last three are the informed prior probability distributions for the remaining parameters. Incubation time was centred to remove correlation between α and β. Where more than one mean or standard deviation are provided, the values refer to light and dark sample and blank incubations in that order. σ is assigned a non-negative exponential prior with rate = 1 by default (McElreath, 2019). A Gaussian prior with mean = 228 µM and standard deviation = 20 µM was chosen for the intercept (α) based on known regional i.e., South Indian Central Water (Woo and Pattiaratchi, 2008) and local (Rose *et al.*, 2012) dissolved O_2. Different slope (β) priors were selected for each incubation type by centring on zero (blanks) or back-transforming rates from similar O_2 evolution experiments on *E. radiata* laterals from our study site (cf. 15–20°C incubation temperature, 120-mL chambers, ~0.22 g dry mass, Fig. 1D, Staehr and Wernberg, 2009; Wernberg *et al.*, 2016). Prior rates of O_2 change (β_p, µM min^{-1}) were back-calculated as

$$\beta_p = \frac{r \times m \times 10^3}{V \times \Delta t \times M} \quad (2)$$

where r is the given mean rate of photosynthesis or respiration (mg O_2 g^{-1} dry mass h^{-1}) for temperatures 15 and 20°C (Fig. 1D), m is the given mean dry mass (0.22 g), V is the given incubation volume (0.12 L), Δt is the reported period (60 min), M is the molar mass of O_2 and 10^3 is the conversion from mM to µM.

For each of the 160 resulting models, prior and posterior probability distributions for β (Fig. S1A) and α (Fig. S1B) were visually compared and means and 98% percentile intervals of μ as well as 98% percentile prediction intervals for predicted observations calculated and visualised over the range of t (Fig. S1C). Posterior probability distributions of both α and β are of interest downstream since $\alpha + \beta \times -\bar{t}$ is an estimate of the initial incubation O_2 (µM) which may affect photosynthesis and respiration. β (µM min^{-1}) posteriors were converted to P_n, R, P_g (mmol O_2 g^{-1} dry mass h^{-1}) and P_d (mmol O_2 g^{-1} dry mass d^{-1}) as

$$P_n, R = \frac{(\beta_{l,d} - \beta_b) \times V \times \Delta t_1}{m \times 10^3} \quad (3)$$
$$P_g = P_n - R$$
$$P_d = (P_n + R) \times \Delta t_2$$

where subscript l, d and b denote light and dark sample and blank incubations from the same measurement group, V is the posterior probability distribution of the mean of empirically derived incubation volume (mean = 175 mL), m is the sample dry mass (g), 10^3 is the conversion from µmol to mmol and Δt is the desired period ($_1$ = 60 min, $_2$ = 12 h). We converted blotted mass to dry mass by multiplying by sample-specific dry-blotted mass ratios to make our rates comparable with the

literature. Note the reversed signs in the last two equations since β_d and therefore R are given as negative values. Importantly, by performing calculations on entire probability distributions rather than central tendencies, all measurement error is propagated. For instance, P_g inherits error from β_l, β_d, β_b and V (Fig. S2, S3). The resulting 57 probability distributions for P were summarised as mean and standard deviation to enable propagation of measurement error to the next series of models (Equation S5).

Confounders were included using multiple linear regression to avoid biased inference. Photosynthesis may be sensitive to a variety of variables, including O_2, temperature, pressure, salinity and resource availability (carbon and nutrients), the latter being a function of the available seawater volume per tissue mass. The O_2 meter automatically corrects for temperature, pressure and salinity, and we normalised by sample mass (Equation S3) and attempted to control temperature using a 20°C controlled temperature room. However, this does not remove the confounding effect of these variables and they deserve incorporation. The posterior probability distribution of initial incubation O_2 for each measurement is readily estimated by $\alpha + \beta \times -\bar{t}$ (Equation S1) and summarised as mean and standard deviation for each value of P (Equation S3). Standardised incubation temperature across each ~5-min measurement (T, °C) was additionally estimated using a simple intercept model as

$$
\begin{aligned}
&T \sim \text{normal}(\text{mean} = \mu, \text{standard deviation} = \sigma) \qquad (4)\\
&\mu = T_\mu\\
&T_\mu \sim \text{normal}(\text{mean} = 0, \text{standard deviation} = 1)\\
&\sigma \sim \text{exponential}(\text{rate} = 1)
\end{aligned}
$$

where the first line is the Gaussian likelihood function describing the response variable, the second describes μ, and the last two are regularising prior probability distributions for the remaining parameters. μ from this model was summarised as mean and standard deviation for each observation of P (Equation S3). Pressure, salinity and sample mass had little to no variability within measurement series, so they were simply summarised as mean for each value of P (Equation S3). Therefore, of the five confounders we identified, only initial incubation O_2 and mean incubation temperature had modellable measurement error, requiring incorporation into the model (McElreath, 2019).

Each of the four tanks assigned to each treatment varied slightly in size, position, water level, sediment depth etc. Partial pooling was employed to incorporate such variation by explicitly modelling the standard deviation across tank slopes. As such, this method estimates a parameter based on other parameters and is therefore called multilevel or hierarchical modelling. Partial pooling is the default for any type of categorical variable (McElreath, 2019) but after fitting a full multilevel model, we chose not to have pooling between treatments because (1) as opposed to tanks, we are not aiming to predict the response for new, unknown treatments and (2) data missingness (see main manuscript for a discussion of this issue) causes the light 15°C treatment to have most observations and skew the slopes of all other treatments towards its value. Under other circumstances this regularisation would be desired, but in our case it just exacerbates the underestimation of the treatment effect caused by non-random missing data. The five initial measurements from 29th June were randomly assigned a tank and treatment prior to the next modelling stage. The uneven number of replicates resulted in an additional observation in the dark 15°C treatment, causing the maximal sample size to be n = 26 rather than n = 25.

Taking all of the above into account, P (mmol O_2 g^{-1} dry mass h^{-1} or mmol O_2 g^{-1} dry mass d^{-1}), inferred from observed P with measurement error ($P_{obs} \pm s_P$), was modelled as a function of detrital age (A, d), numerically coded versions of the four-level treatment variable (Tr, dark 15°C, light 15°C, light 20°C and light 25°C) and the 16-level tank variable (Ta) and standardised versions of the incubation confounders initial O_2 with measurement error ($O_{obs} \pm s_O$, µM), mean incubation temperature with measurement error ($T_{obs} \pm s_T$, °C), mean pressure (Pr, hPa), salinity (S, ‰) and sample dry mass (M, g) as

$$
\begin{aligned}
&P \sim \text{normal}(\text{mean} = \mu, \text{standard deviation} = \sigma) \qquad (5)\\
&\mu = \alpha + (\beta_{Tr} + \beta_{Ta}) \times A + \beta_O \times O + \beta_T \times T +\\
&\quad \beta_{Pr} \times Pr + \beta_S \times S + \beta_M \times M\\
&\beta_{Ta} = z \times \tau\\
&P_{obs} \sim \text{normal}(\text{mean} = P, \text{standard deviation} = s_P)\\
&O_{obs} \sim \text{normal}(\text{mean} = O, \text{standard deviation} = s_O)\\
&T_{obs} \sim \text{normal}(\text{mean} = T, \text{standard deviation} = s_T)\\
&O, T \sim \text{normal}(\text{mean} = 0, \text{standard deviation} = 1)\\
&\alpha \sim \text{normal}(\text{mean} = 0.05, 0.07, 0.27, \text{standard deviation} = 0.02, 0.02, 0.2)\\
&\beta_{Tr} \sim \text{normal}(\text{mean} = -0.001, -0.001, -0.01, \text{standard deviation} = 0.001, 0.001, 0.02)\\
&\beta_O, \beta_T, \beta_P, \beta_S, \beta_M \sim \text{normal}(\text{mean} = 0, \text{standard deviation} = 0.01, 0.01, 0.2)\\
&z \sim \text{normal}(\text{mean} = 0, \text{standard deviation} = 1)\\
&\sigma, \tau \sim \text{exponential}(\text{rate} = 1)
\end{aligned}
$$

where the first line is the Gaussian likelihood function describing the response variable, the second is the linear model describing μ, the third enables partial pooling of tank slopes without the hierarchical priors typical of multilevel models by estimating their *z*-score, the next four are priors for every observation in variables with measurable error, and the last five are priors for all parameters. Where more than one mean or standard deviation are provided, the values refer to net, gross and daily net photosynthesis in that order. Intercept priors were selected from previous *E. radiata* photosynthesis rates at 15–20°C (Fig. 1D, Staehr and Wernberg, 2009; Wernberg *et al.*, 2016), and converted using dry to blotted mass ratios from our study. Slope priors were derived by averaging the only similar parameters available in the literature across species (Wright *et al.*, 2022).

To complete the picture, we incorporated additional information contained in the unmeasurable disintegrated samples by calculating three binomial variables (see *Data analysis and visualisation* in main manuscript). Each probability (*P*, 0 or 1) was modelled as a function of detrital age (*A*, d), numerically coded versions of the four-level treatment variable (*Tr*) and the 16-level tank variable (*Ta*) as

$$
\begin{aligned}
&P \sim \text{binomial}(\text{number} = 1, \text{probability} = p) \qquad (6)\\
&\text{logit}(p) = \alpha + (\beta_{Tr} + \beta_{Ta}) \times A\\
&\beta_{Ta} = z \times \tau\\
&\alpha \sim \text{normal}(\text{mean} = 5, \text{standard deviation} = 2.5)\\
&\beta_{Tr} \sim \text{normal}(\text{mean} = -0.1, \text{standard deviation} = 0.2)\\
&z \sim \text{normal}(\text{mean} = 0, \text{standard deviation} = 1)\\
&\tau \sim \text{exponential}(\text{rate} = 1)
\end{aligned}
$$

where the first line is the Bernoulli likelihood function describing the response variable, the second is the linear model describing logit-transformed *p*, i.e., log odds, the third enables partial pooling of tank slopes without the hierarchical priors typical of multilevel models by estimating their *z*-score and the last four are priors. The above priors were chosen since $\alpha = 5$ on the log odds scale translates to almost near certainty of survival, which is sensible for $A = 0$, and $\beta_{Tr} = -0.1$, which corresponds to the logistic rate *k* (log odds d^{-1}) in this case, provides an indication of the expected negative slope, i.e., logistic decay. Since the intercept is already known to lie near 1 on the probability scale, time at which $p = 0.5$ is more meaningful to our model interpretation. This is given by the inflection point of the sigmoid curve on the probability scale (μ), the probability distributions of which was calculated as $\mu = \frac{\alpha}{\beta}$. Note that μ (d) is a quotient distribution and therefore does not have a defined mean or standard deviation, so the quotient of the means of α and β was used as a close proxy for the mean of μ.

References

McElreath R. **2019**. *Statistical rethinking*^{2}*: a Bayesian course with examples in R and Stan. Second Edition*. Boca Raton: Chapman & Hall/CRC.

Rose TH, Smale DA, Botting G. **2012**. The 2011 marine heat wave in Cockburn Sound, southwest Australia. *Ocean Science* **8**: 545–550.

Staehr PA, Wernberg T. **2009**. Physiological responses of *Ecklonia radiata* (Laminariales) to a latitudinal gradient in ocean temperature. *Journal of Phycology* **45**: 91–99.

Wernberg T, de Bettignies T, Joy BA, Finnegan PM. **2016**. Physiological responses of habitat-forming seaweeds to increasing temperatures. *Limnology and Oceanography* **61**: 2180–2190.

Woo M, Pattiaratchi C. **2008**. Hydrography and water masses off the western Australian coast. *Deep Sea Research Part I: Oceanographic Research Papers* **55**: 1090–1104.

Wright LS, Pessarrodona A, Foggo A. **2022**. Climate-driven shifts in kelp forest composition reduce carbon sequestration potential. *Global Change Biology* **28**: 5514–5531.

Supplementary figures

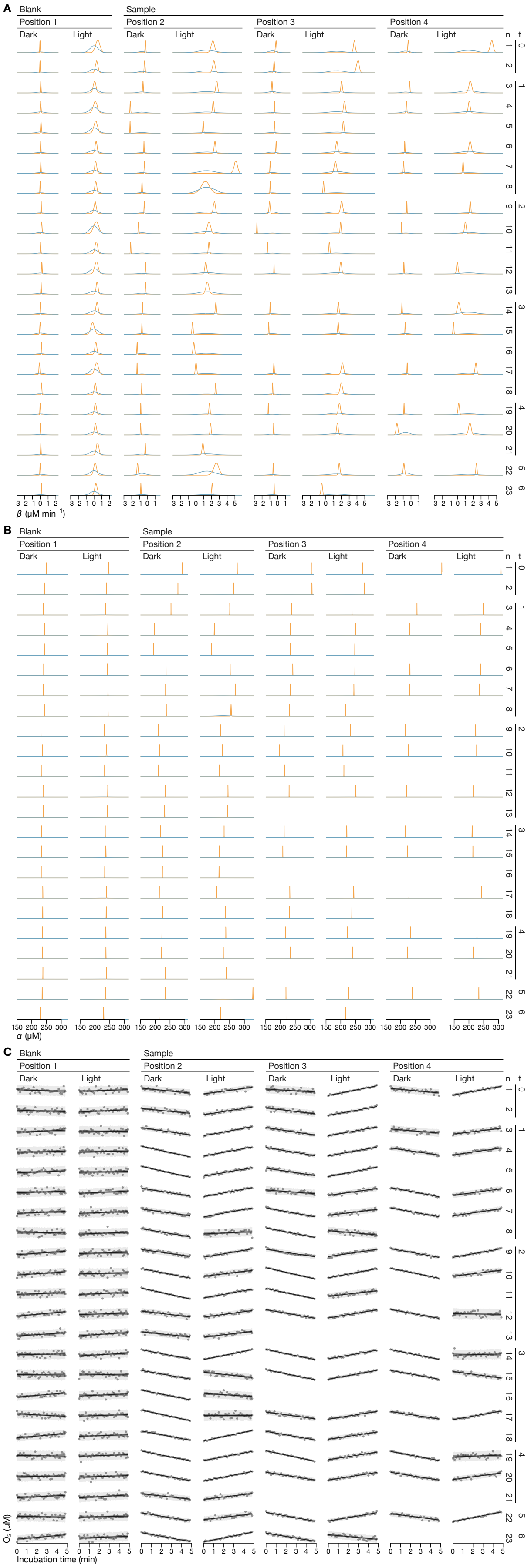

Figure S1. Dissolved O_2 model (Equation S1) parameters. Prior (blue) and posterior (orange) probability distributions of β (**A**) and α (**B**) in Equation S1. **C**, The combination of both parameters yields an estimate of μ across the predictor variable t_c (Equation S1). t_c has here been replaced by the non-centred t for visualisation purposes. Lines and dark ribbons are means and 98% posterior probability intervals for μ while light ribbons are 98% posterior probability intervals for O (Equation S1). In all panels, incubations are stratified by their position on the four-position magnetic stirrer (counting row-wise from left to right), the measurement round (n) and the timepoint (t).

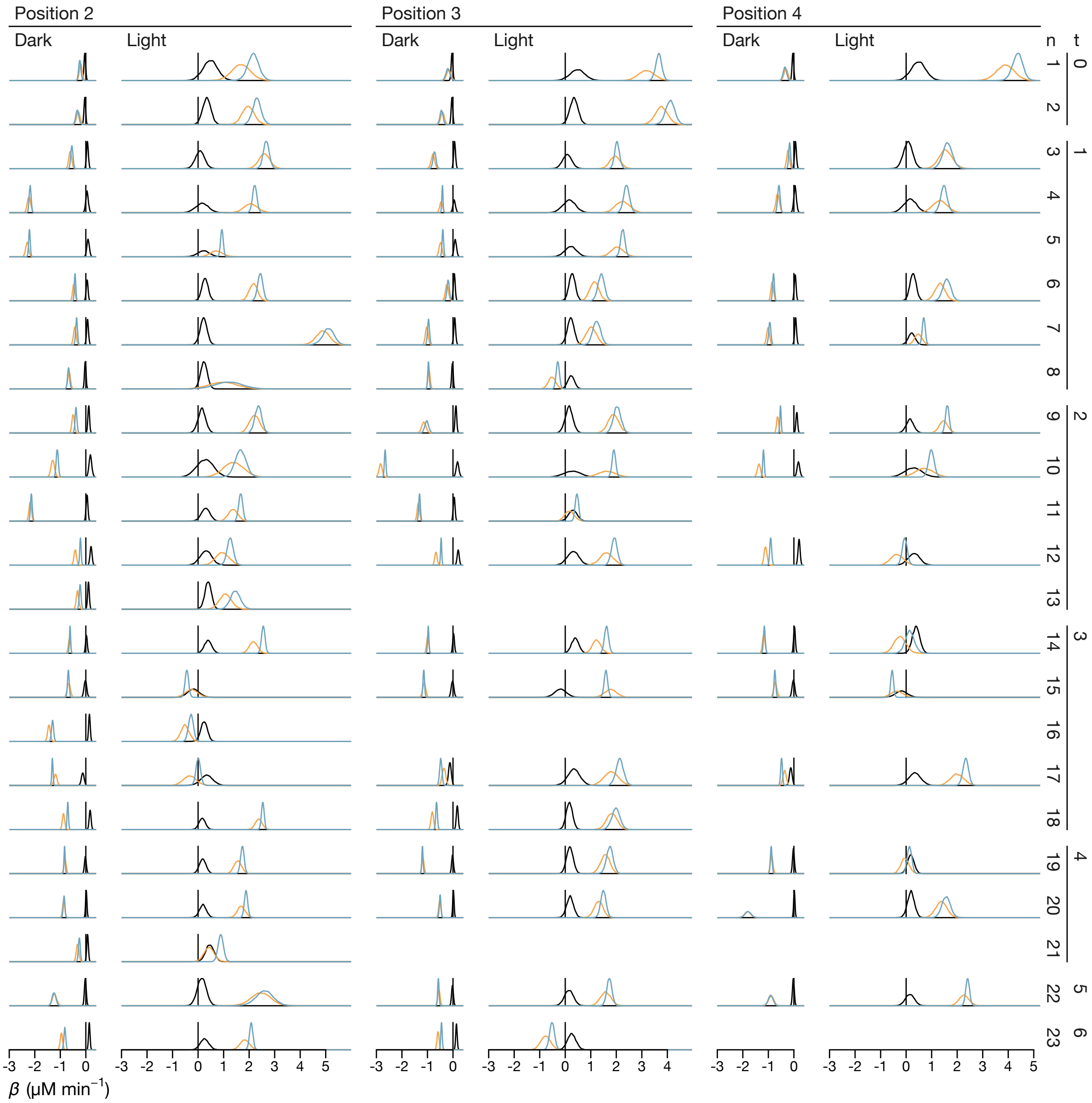

Figure S2. Blank correction of slopes (Equation S3). Posterior probability distributions of β (Equation S1) are shown for blanks (black), samples (blue) and samples after subtraction of the blank by measurement round (orange). Note that variation is added by blank subtraction. Incubations are stratified by their position on the four-position magnetic stirrer (counting row-wise from left to right), the measurement round (n) and the timepoint (t).

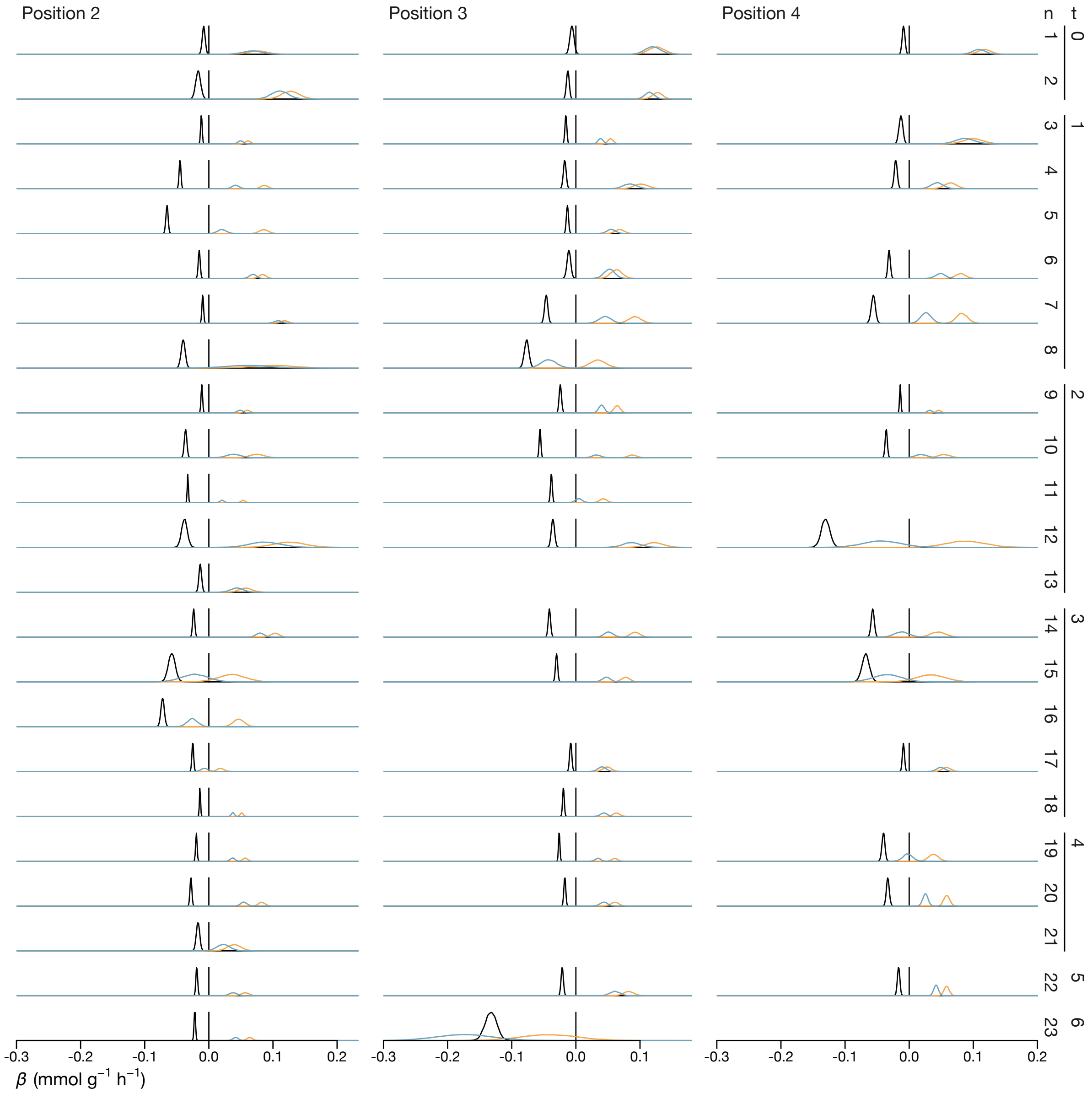

Figure S3. Calculation of gross photosynthesis (Equation S3). Posterior probability distributions of β after blank correction (Fig. S2) and normalisation by volume and sample dry mass (Equation S3) are shown for respiration (black), net photosynthesis (blue) and gross photosynthesis (orange). Note that variation is added by subtracting respiration. Incubations are stratified by their position on the four-position magnetic stirrer (counting row-wise from left to right), the measurement round (n) and the timepoint (t).

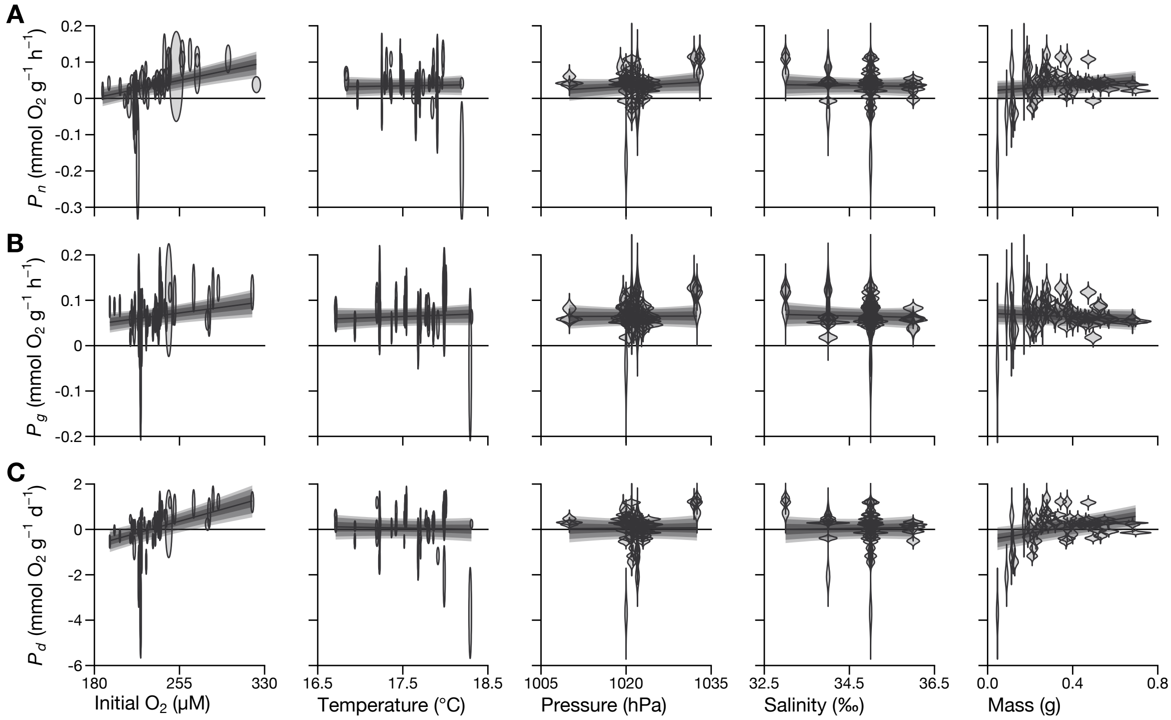

Figure S4. Effect of confounding variables associated with incubation on net (**A**), gross (**B**) and daily (24-h) net (**C**) detrital photosynthesis of *Ecklonia radiata*, given per gram of dry mass. Ellipses (first two panels) are bivariate posterior probability distributions for each observation, derived from β (Equation S1) via conversion (Equation S3) and $\alpha + \beta \times -\bar{t}$ (Equation S1) or μ (Equation S4) for initial O_2 and temperature respectively. Violins (last three panels) are posterior probability distributions for each observation, derived from β (Equation S1) via conversion (Equation S3). Lines and intervals are means and 50, 80 and 90% posterior probability intervals for μ (Equation S5) at mean detrital age $\bar{A}$.

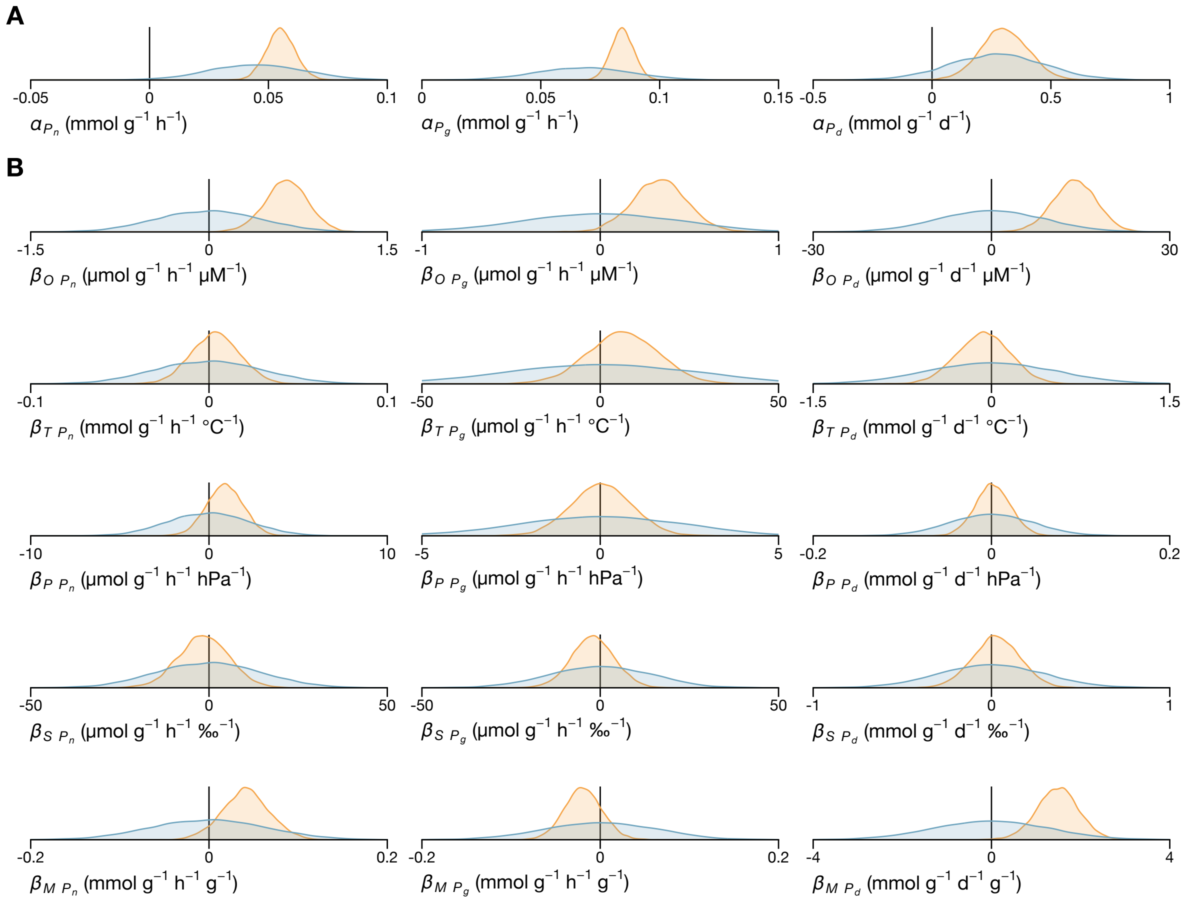

Figure S5. Linear photosynthesis model (Equation S5) parameters. Prior (blue) and posterior (orange) probability distributions of α (**A**) and β_O, β_T, β_P, β_S and β_M (**B**) in Equation S5.

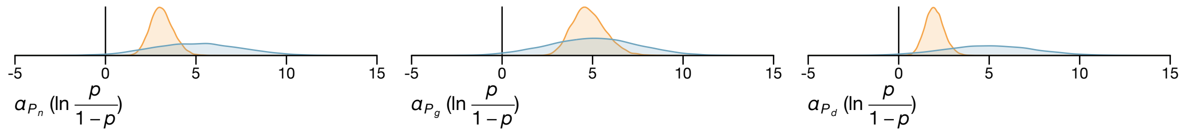

Figure S6. Binomial photosynthesis model (Equation S6) parameters. Prior (blue) and posterior (orange) probability distributions of α in Equation S6.
